# Genome-wide analysis of differentially expressed mRNAs, lncRNAs, and circRNAs in chicken bursae of Fabricius during infection with very virulent infectious bursal disease virus

**DOI:** 10.1186/s12864-020-07129-1

**Published:** 2020-10-19

**Authors:** Xuewei Huang, Junyan Zhang, Zengsu Liu, Meng Wang, Xiaolong Fan, Li Wang, Han Zhou, Yanping Jiang, Wen Cui, Xinyuan Qiao, Yigang Xu, Yijing Li, Lijie Tang

**Affiliations:** 1 College of Veterinary Medicine, Northeast Agricultural University, Changjiang Road No. 600, Xiang Fang District, Harbin, People’s Republic of China; 2 Heilongjiang Key Laboratory for Animal Disease Control and Pharmaceutical Development, Harbin, People’s Republic of China

**Keywords:** Infectious bursal disease virus, Bursa of Fabricius, mRNAs, lncRNAs, circRNAs

## Abstract

**Background:**

Infectious bursal disease virus (IBDV) causes acute, highly contagious, immunosuppressive, and lethal infectious disease in young chickens and mainly infects the bursa of Fabricius (BF). To investigate interactions between IBDV and its host, RNA sequencing was applied to analyze the responses of the differentially expressed transcriptional profiles of BF infected by very virulent IBDV (vvIBDV).

**Results:**

In total, 317 upregulated and 94 downregulated mRNAs were found to be significantly differentially expressed in infected chickens, compared to controls. Long non-coding RNA (lncRNA) and circular RNA (circRNA) alterations were identified in IBDV-infected chickens, and significantly different expression was observed in 272 lncRNAs and 143 circRNAs. Gene Ontology and Kyoto Encyclopedia of Genes and Genomes pathway enrichment analyses were performed to assess the functions of significantly dysregulated genes, which showed that the JAK-STAT signaling pathway, the NOD-like receptor signaling pathway, and apoptosis may be activated by IBDV infection. We predicted interactions between differentially expressed genes and produced lncRNA-mRNA and circRNA-miRNA-mRNA regulator network.

**Conclusions:**

The present study identified the expression profiles of mRNAs, lncRNAs, and circRNAs during vvIBDV infection and provides new insights into the pathogenesis of IBDV and antiviral immunity of the host.

## Background

Infectious bursal disease virus (IBDV), a non-enveloped double-stranded RNA virus, is a member of the family *Birnaviridae*; it can cause acute, highly contagious, and immunosuppressive disease in chickens aged 3–6 weeks, leading to high mortality and considerable economic losses [1, 2]. Serotype-I and -II strains of IBDV are recognized, and serotype I, which includes attenuated, classically virulent, and very virulent (vv) variants, can cause different degrees of pathogenicity and mortality in chickens [3]. IBDV predominantly targets the precursors of B lymphocytes, particularly those in the bursa of Fabricius (BF), an important immune organ, the infection of which may lead to B lymphocyte depletion and BF deterioration [4, 5]. The severe immunosuppression caused by IBDV increases the susceptibility of chickens to other infectious diseases and reduces immune responses to vaccination [6]. Therefore, elucidating the interactions between IBDV and its host is a matter of urgency to determine effective strategies for preventing and controlling IBDV infections.

High-throughput sequencing technology and proteomic approaches have been used for this purpose, and various types of non-coding RNAs (ncRNAs) are also gaining increasing attention in this regard [7], including long non-coding RNAs (lncRNAs) [8] and circular RNAs (circRNAs) [9]. lncRNAs are longer than 200 nucleotides in length and can regulate gene expression at various levels, including epigenetics and transcriptional and post-transcriptional regulation. lncRNAs typically occur at low abundance and are frequently not conserved between species [10]. Moreover, lncRNAs have been displayed to affect viral replication by regulating the expression of antiviral-related key genes [11–13], indicating that lncRNA might play a crucial role in antiviral responses. CircRNAs form covalently closed continuous loops, which have been observed to be widely expressed in plants [14] and animals [15, 16]. More importantly, studies have demonstrated that circRNAs influence viral infection by inhibiting viral factors [17, 18]. Therefore, it is important to identify lncRNAs and circRNAs as well as their targets to understand the dynamics of gene regulation and effectively control the occurrence and development of disease.

Although many studies have been conducted to assess the effects of host lncRNAs on IBDV infection and its underlying molecular mechanisms [11, 19–21], the genome-wide expression effects and functional roles of lncRNAs and circRNAs during vvIBDV infection have not been examined so far. We investigated the expression profiles of mRNAs, lncRNAs, and circRNAs associated with vvIBDV infection of chicken’ BF and constructed lncRNA-mRNA and circRNA-miRNA-mRNA co-expression networks, which may provide valuable information for new therapeutic approaches to control this disease.

## Results

### Identification of lncRNAs and circRNAs in chicken bursa tissue

**Fig. 1:**
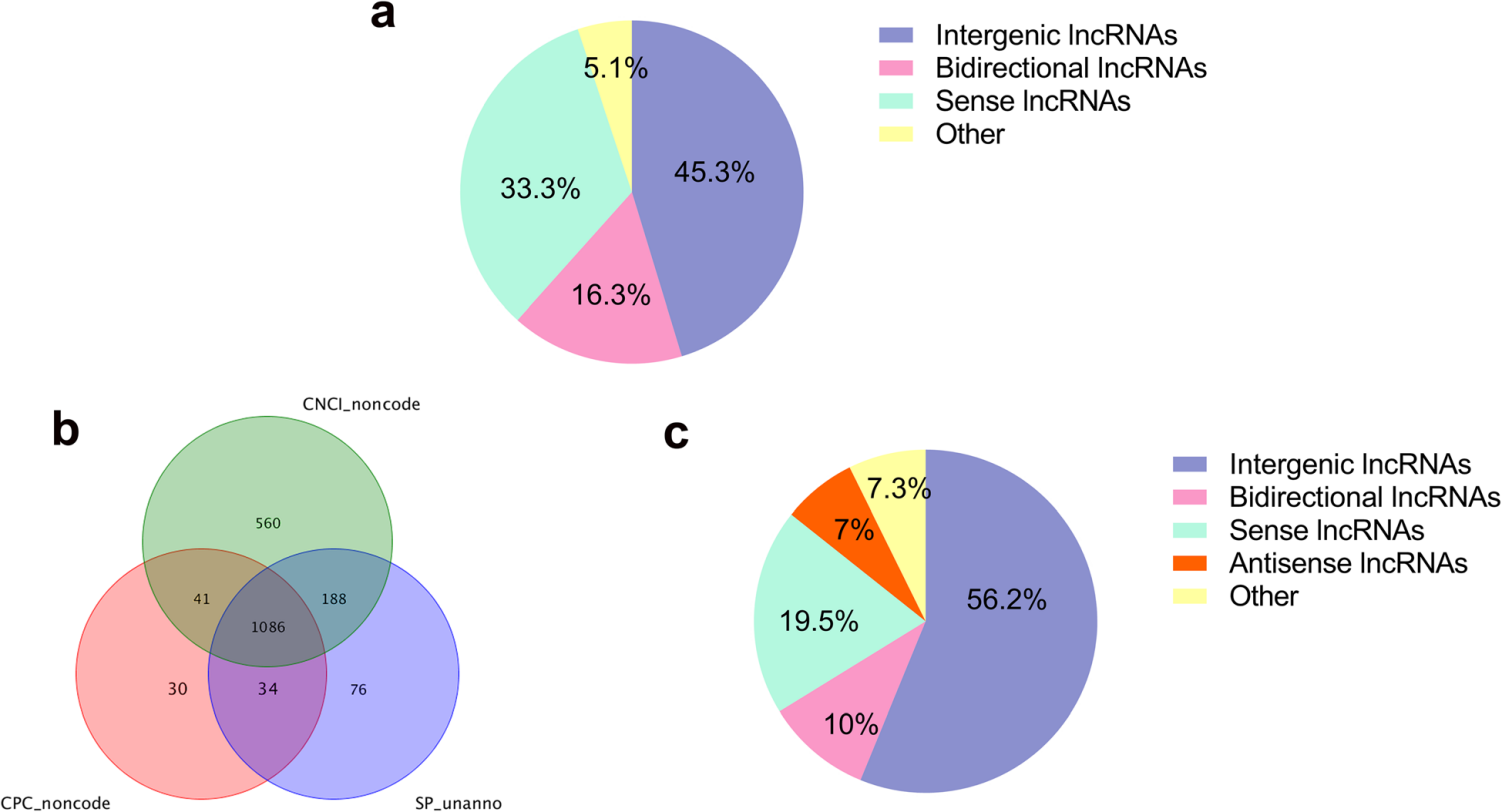
Screening and classification of lncRNAs in chicken BF. **a** The novel lncRNAs were mainly classified as intergenic lncRNAs, bidirectional lncRNAs, and sense lncRNAs. **b** CNCI, CPC, and the SwissProt database were used to analyze the coding potential of the novel lncRNAs. RNAs identified by all three analytical tools were chosen as candidate lncRNAs. **c** The novel lncRNAs were mainly classified as intergenic lncRNAs, sense lncRNAs, bidirectional lncRNAs, and antisense lncRNAs

In total, 589,776,342 raw reads were obtained from the control and IBDV-infected bursa tissues. After data filtering and quality control, 584,284,990 clean reads of high quality were retained, and the proportion of clean reads ranged from 98.99 to 99.13% (Additional file 1: Table S1). Subsequently, reads mapping to ribosomal RNA (rRNA) were removed, and the proportion of retained reads ranged from 86.69 to 99.54% (Additional file 2: Table S2). After removing the rRNA, clean reads were mapped to the chicken reference genome (Table 1). The transcripts were screened using Cufflinks software (v. 2.1.1) [22] based on the location of the transcripts on the reference genome, a transcript length of ≥200 bp, and an exon count of ≥2. In total, 4324 known lncRNAs transcripts (Additional file 3) were obtained from chicken bursa tissue, including 1957 (45.3%) intergenic lncRNAs, 706 (16.3%) bidirectional lncRNAs, 1442 (33.3%) sense lncRNAs, and (5.1%) 219 lncRNAs of other types. Nevertheless, intronic lncRNA and antisense lncRNA were not detected in our study. Moreover, 1086 novel lncRNAs were screened based on three protein-coding potential software analyses (Fig. 1a; Additional file 4). The 1086 novel lncRNAs comprised 610 (56.2%) intergenic lncRNAs, 212 (19.5%) sense lncRNAs, 109 (10.0%) bidirectional lncRNAs, 76 (7.0%) antisense lncRNAs, and 79 (7.3%) lncRNAs of other types (Fig. 1b). No intronic lncRNAs were detected in the current study. Besides, anchor reads were mapped to the chicken reference genome, 7808 novel circRNAs were identified in the study (Additional file 5; Table 2).

**Table 1:** High quality clean reads compared with the reference genome

Sample	Total reads	Unmapped reads	Unique mapped reads	Multiple mapped reads	Mapping ratio
CK-1	63,612,526	6,924,650 (10.89%)	56,364,212 (88.61%)	323,664 (0.51%)	89.11%
CK-2	102,560,584	12,194,879 (11.89%)	89,682,285 (87.44%)	683,420 (0.67%)	88.11%
CK-3	86,188,102	10,670,110 (12.38%)	74,975,168 (86.99%)	542,824 (0.63%)	87.62%
LJ-1	91,490,442	11,614,304 (12.69%)	79,452,098 (86.84%)	424,040 (0.46%)	87.31%
LJ-2	147,560,076	17,994,743 (12.19%)	128,778,345 (87.27%)	786,988 (0.53%)	87.81%
LJ-3	80,368,368	10,384,074 (12.92%)	69,600,696 (86.60%)	383,598 (0.48%)	87.08%

**Table 2:** Anchor reads compared with the reference genome

Sample	Anchors number	Mapped anchors	Mapping ratio
CK-1	13,849,300	10,972,238	79.23%
CK-2	24,389,758	19,806,822	81.21%
CK-3	21,340,220	17,321,681	81.17%
LJ-1	23,228,608	18,759,901	80.76%
LJ-2	35,989,486	29,433,145	81.78%
LJ-3	20,768,148	16,500,270	79.45%

### Differentially expressed profiles of mRNAs, lncRNAs, and circRNAs

**Fig. 2:**
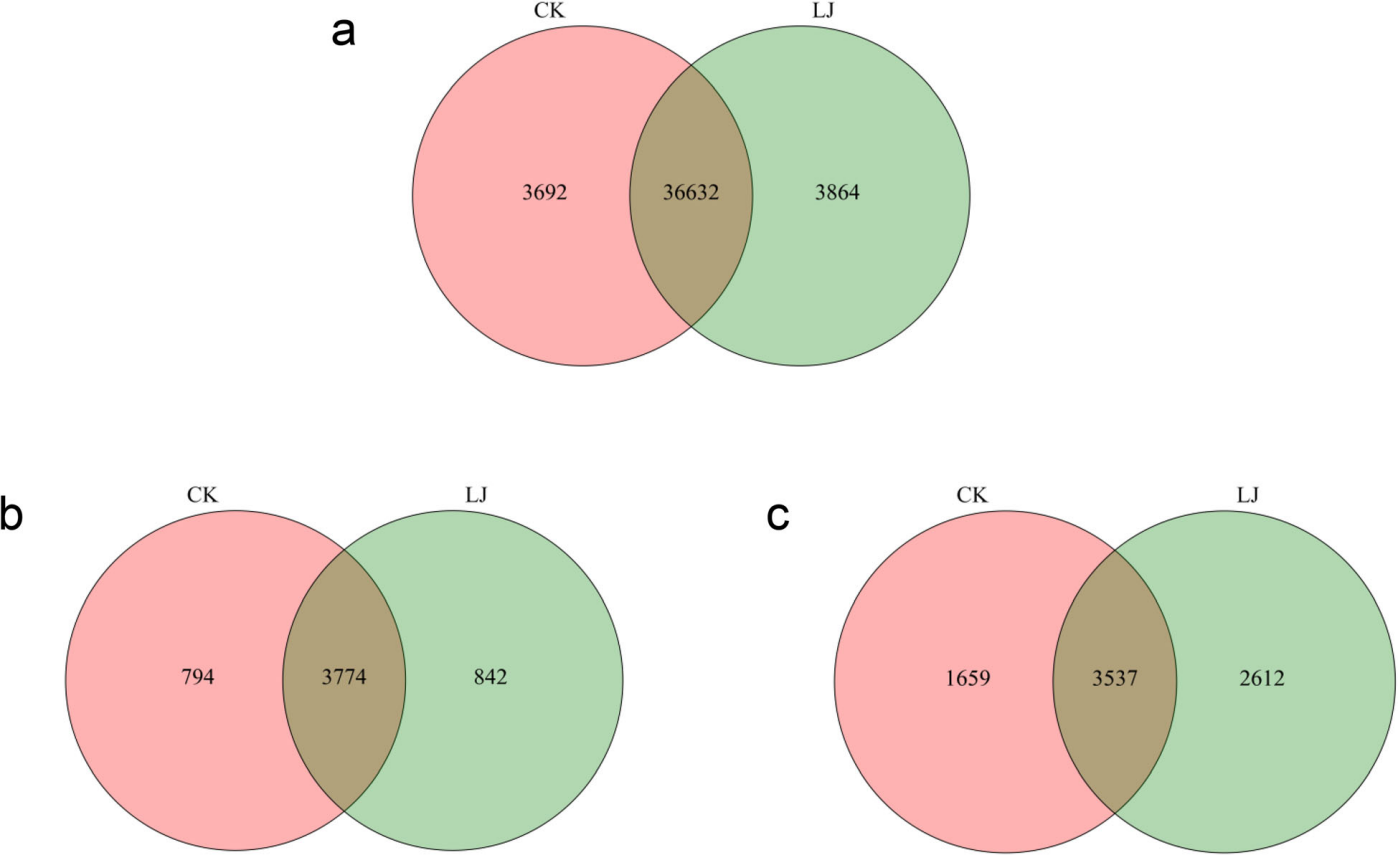
Venn diagram showing the number of overlapping genes in the IBDV-infected group and the control group. **a** mRNAs, **b** lncRNAs, and **c** circRNAs

**Fig. 3:**
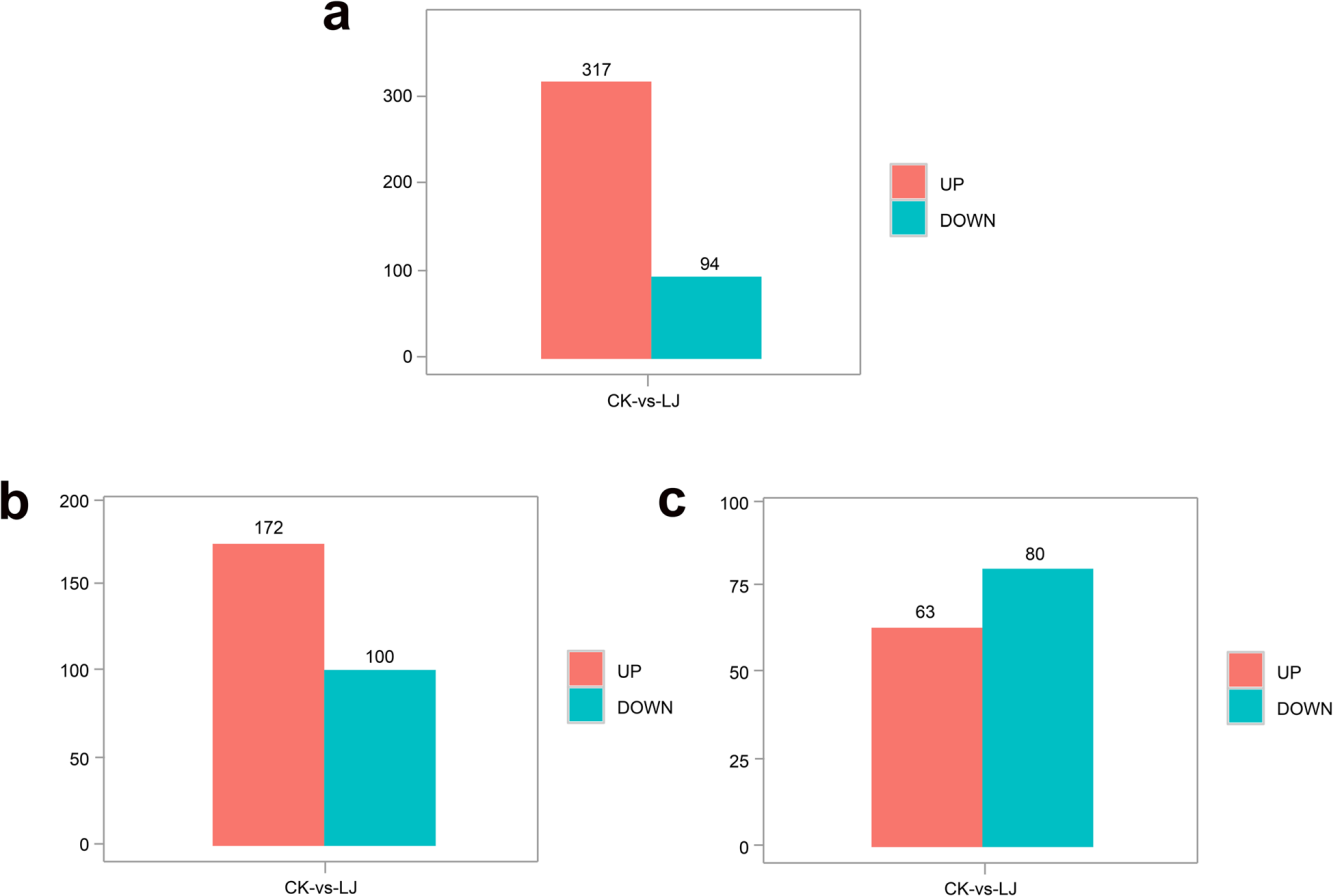
Histogram of the differentially expressed mRNAs (**a**), lncRNAs (**b**) and circRNAs (**c**) in the two groups. The red and green columns indicate the significantly upregulated and downregulated genes (*p* < 0.05), respectively

**Fig. 4:**
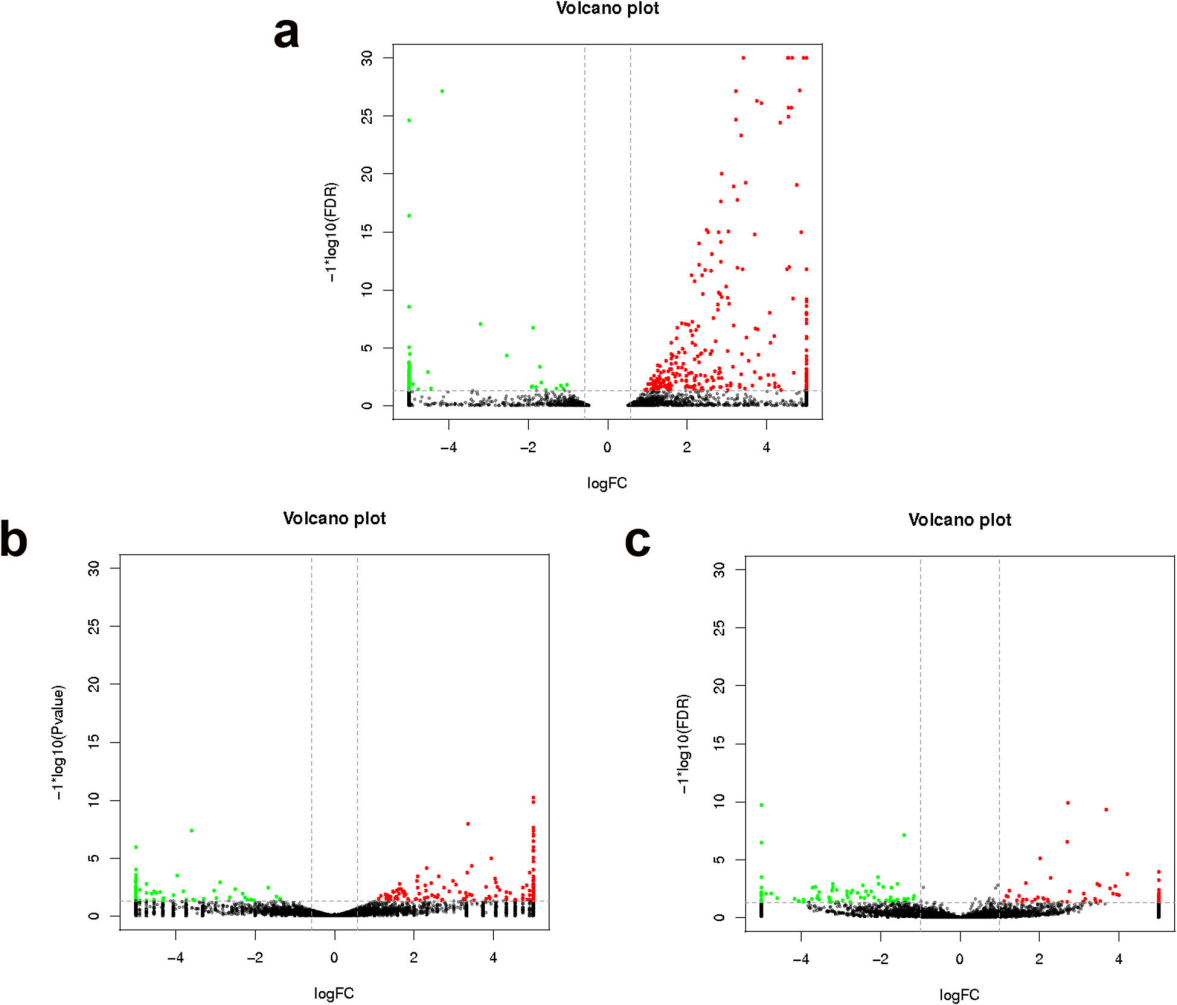
Volcano plots of the differentially expressed mRNAs (**a**), lncRNAs (**b**), and circRNAs (**c**). The red and green dots indicate the significantly upregulated and downregulated genes (*p* < 0.05), respectively. The black dots indicate the genes that were not significantly differentially expressed (*p* > 0.05)

**Fig. 5:**
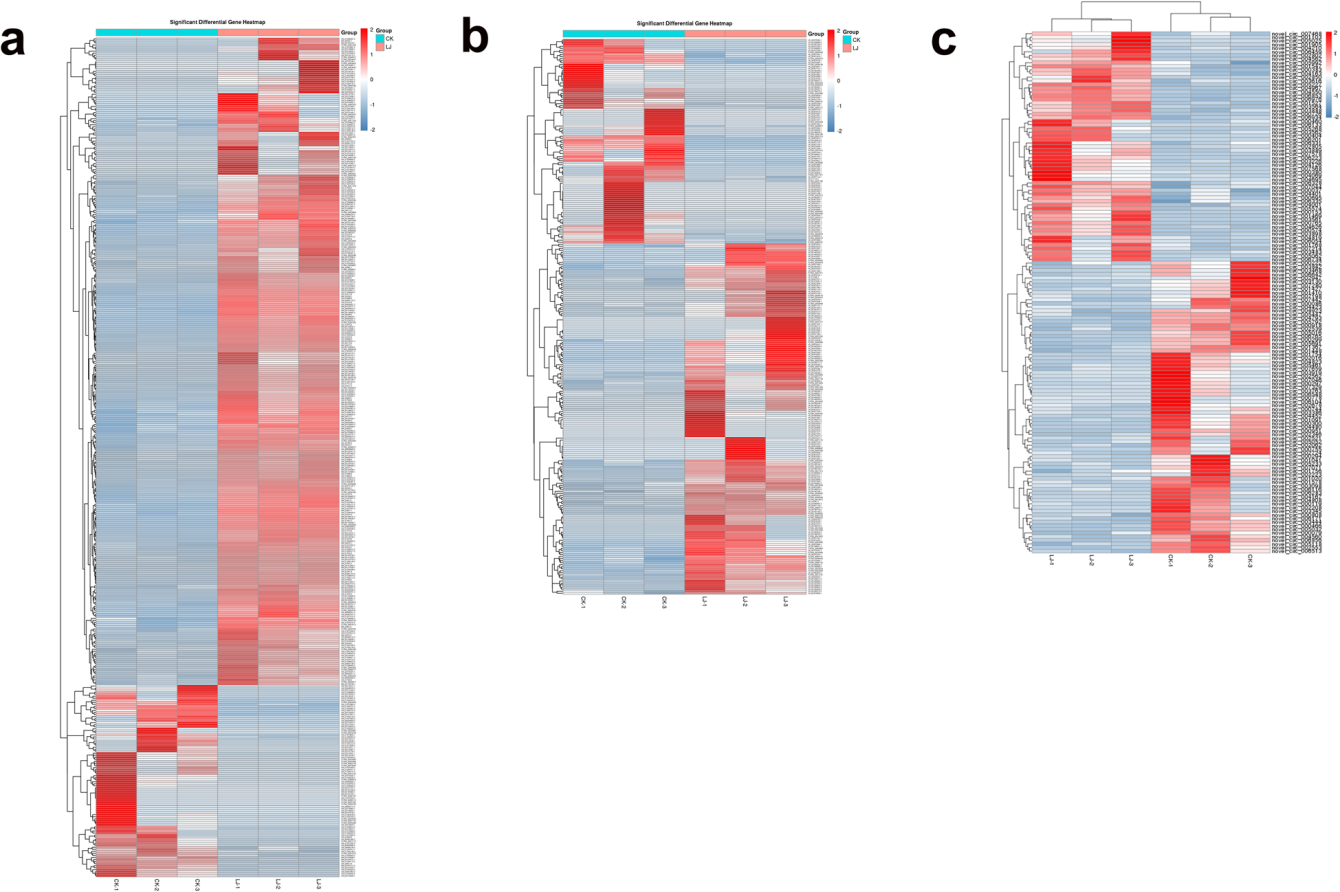
Heatmap of differentially expressed mRNAs (**a**), lncRNAs (**b**), and circRNAs (**c**). The coloration gradient from blue to red indicates low to high expression level. All biological replicates were pooled to identify the DEGs based on a threshold fold change > 2 (mRNAs and circRNAs) or fold change > 1.5 (lncRNAs) at *p* < 0.05

The number of mRNAs, lncRNAs, and circRNAs shared between the infection group and the control group is shown in Fig. 2. Based on the criteria *p* < 0.05 and fold change > 1.5 or 2, 411 mRNAs (Additional file 6), 272 lncRNAs (Additional file 7), and 143 circRNAs (Additional file 8) were considered differentially expressed (Table 3). Moreover, 317 mRNAs were upregulated and 94 were downregulated in the treatment group (Figs. 3a and 4a). Moreover, 172 upregulated and 100 upregulated lncRNAs were identified (Figs. 3b and 4b), and 63 and 80 circRNAs were upregulated and downregulated, respectively (Figs. 3c and 4c). Differentially expressed mRNAs, lncRNAs, and circRNAs were used for cluster analysis. A heat map indicated that the control and IBDV-infected individuals produced two separate clusters (Fig. 5). The expression patterns of the mRNAs, lncRNAs, and circRNAs thus differed between the two groups, suggesting that the differentially expressed genes (DEGs) in the chicken bursa tissue infected with IBDV were significantly different from those in the non-infected tissue.

**Table 3:** The number of differentially expressed mRNA and lncRNA in IBDV treated group

Genes	Up-regulated	Down-regulated	Total
mRNA	317	94	411
lncRNA	172	100	272
circRNA	80	63	143

### Comparison of mRNA and lncRNA characteristics

**Fig. 6:**
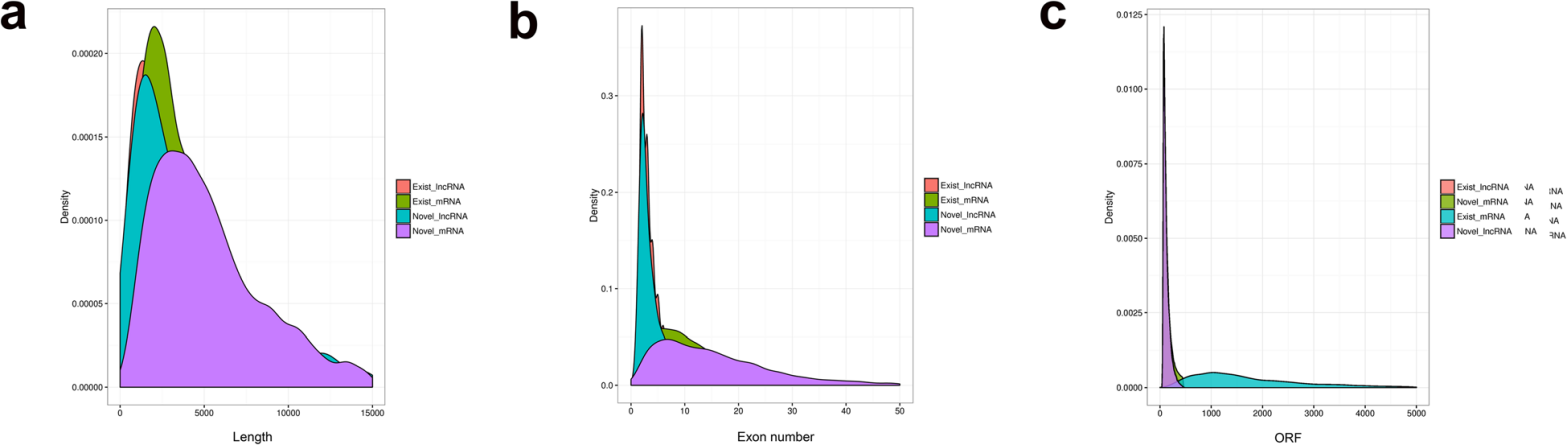
Comparison of mRNA and lncRNA characteristics. **a** Number of exons in the mRNAs and lncRNAs. **b** Distribution of transcript lengths in the mRNAs and lncRNAs. The horizontal axis indicates the length of the transcripts, and the vertical axis indicates the abundance. **c** Number of open reading frames (ORFs) in the mRNAs and lncRNAs

In total, 44188 mRNAs and 5410 lncRNAs were identified in all samples. To examine the differences in the mRNAs and lncRNAs, genetic structure and sequence conservation was compared, and the distribution of the transcript length of the lncRNAs differed slightly from that of the mRNAs (Fig. 6a). However, there were fewer exons in the lncRNAs than in the mRNAs (Fig. 6b). In addition, the open reading frames of the lncRNAs were shorter than those of the mRNAs (Fig. 6c).

### Functional annotation of DEGs

**Fig. 7:**
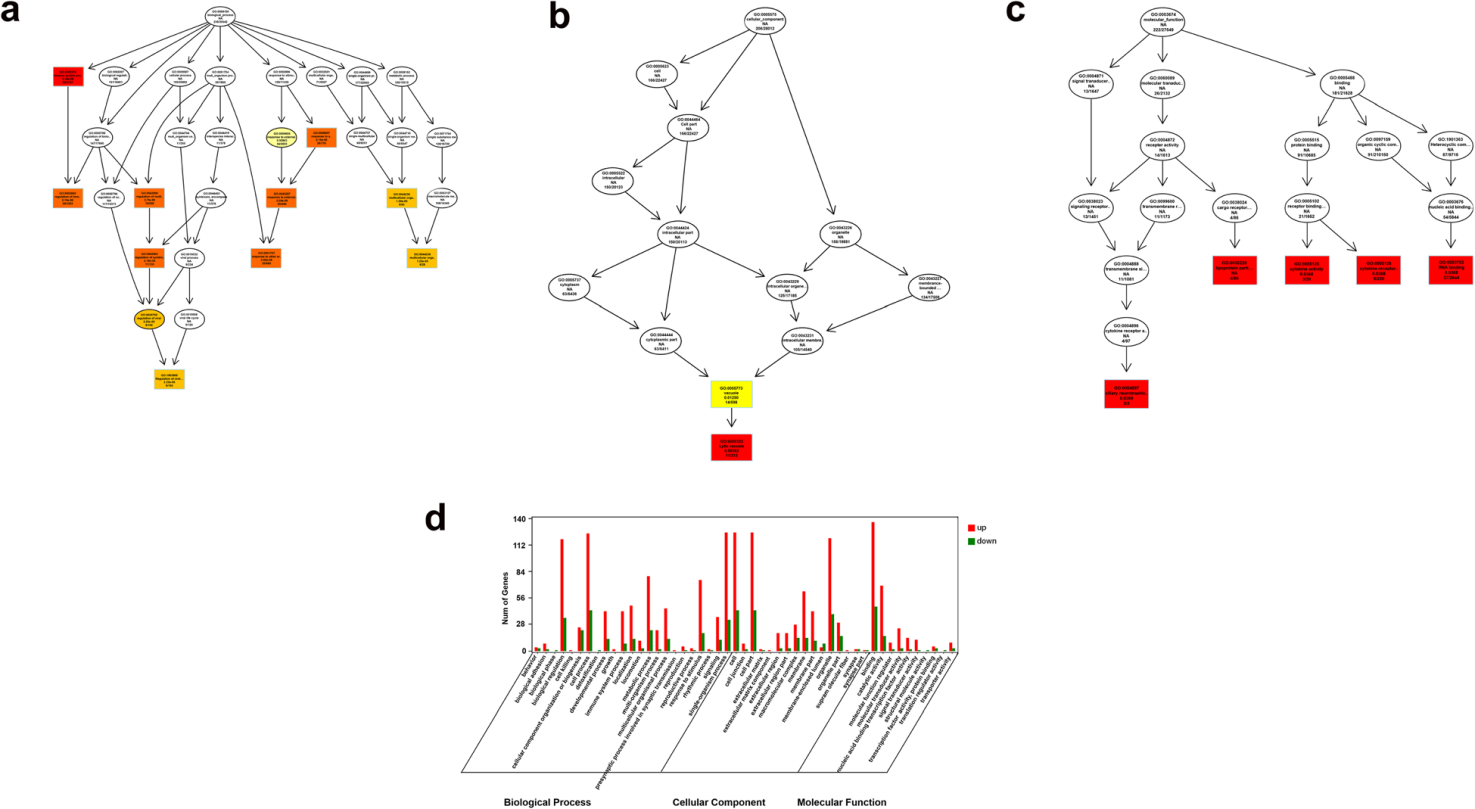
Gene ontology (GO) analysis of the differentially expressed mRNAs in IBDV-infected chicken BF. **a-c** Directed acyclic graph showing the significantly enriched biological processes, cellular components, and molecular functions of the differentially expressed mRNAs. **d** Number of genes in GO terms are shown in the histogram

**Fig. 8:**
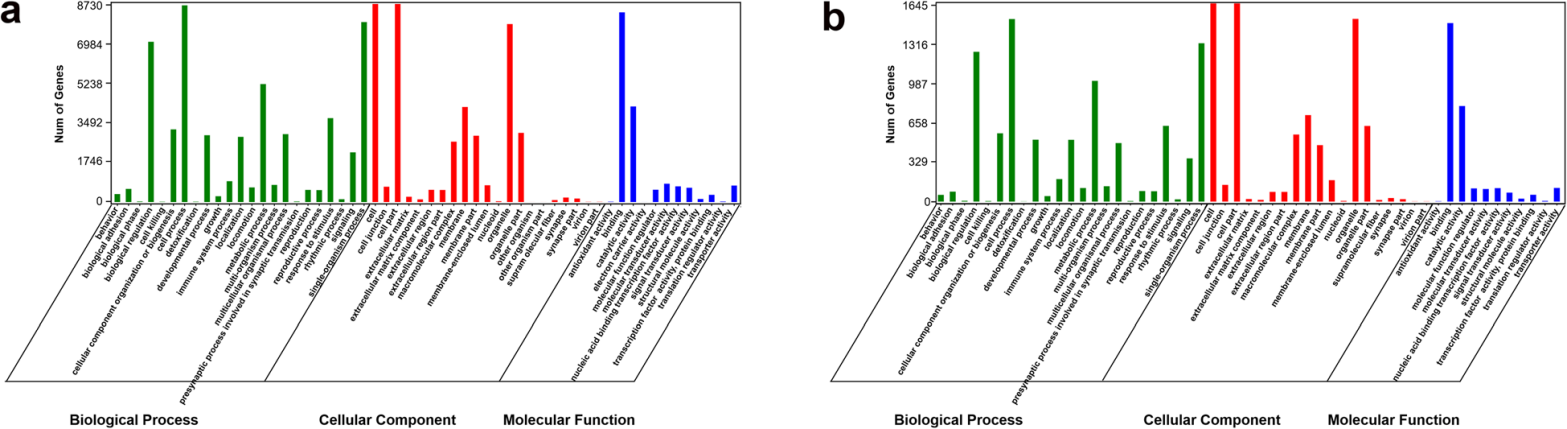
Gene ontology analysis of the differentially expressed lncRNAs (**a**) and circRNAs (**b**) in the two groups. The green, red, and blue column indicate biological processes, cellular components, and molecular functions, respectively

Gene Ontology (GO) and Kyoto Encyclopedia of Genes and Genomes (KEGG) pathway analyses were performed to analyze the 411 differentially expressed mRNAs and the target genes of the differentially expressed lncRNAs and circRNAs to examine the functions of gene dysregulation during IBDV infection. GO enrichment of the biological processes (BPs), cellular components (CCs), and molecular functions (MFs) of the differentially expressed mRNAs, lncRNAs, and circRNAs is shown in Figs. 7 and 8. BPs associated with the enriched mRNAs predominantly included biological regulations, cellular processes, and single-organism processes; enriched mRNA CCs included cells, cell parts, and organelles; and significantly enriched MFs included binding and catalytic activities (Fig. 7; Additional file 9). Based on the GO analyses of the differentially expressed lncRNA target genes, the most enriched BPs were cellular processes, biological regulation, and single-organism processes; the most enriched CCs were cells, cell parts, and organelles; and the most enriched MFs were binding and catalytic activity (Fig. 8a). GO enrichment analysis was performed for the antisense, cis, and trans roles of the target genes of the lncRNA, showing that target genes were also mainly enriched in cellular processes, cells, and binding (Additional file 10: Figure S1). The circRNA results were consistent with those of the lncRNAs (Fig. 8b).

**Fig. 9:**
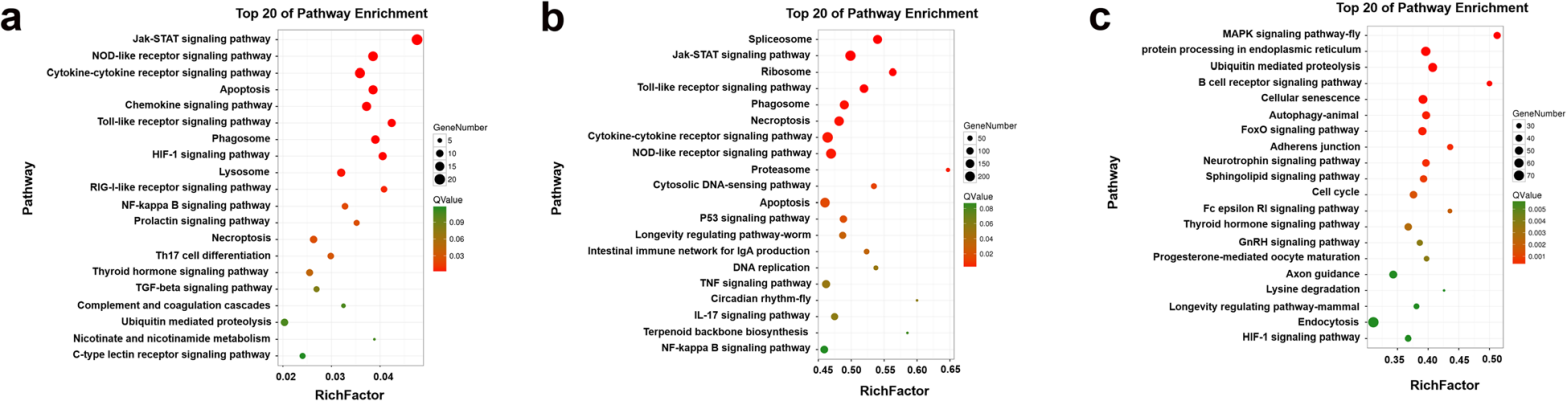
Kyoto Encyclopedia of Genes and Genomes pathway enrichment of the differentially expressed mRNAs (**a**), lncRNAs (**b**), and circRNAs (**c**) in the two groups. The vertical axis indicates the pathways, and the horizontal axis represents the Rich factor. The dot size indicates the number of DEGs in the pathway, and the coloration corresponds to the Q-value range

KEGG is the main public pathway-related database, and it has been used previously to determine the significantly enriched pathways of dysregulated gene products during IBDV infection [23, 24]. The top 20 pathways associated with the mRNAs, lncRNAs, and circRNAs are shown in Fig. 9. The results show that mRNAs differentially expressed owing to IBDV infection were associated with the JAK-STAT signaling pathway, the NOD-like receptor signaling pathway, and the cytokine-cytokine receptor interaction signaling pathway, among others (Fig. 9a; Additional file 11). According to the KEGG analyses of the target genes of the differentially expressed lncRNAs, spliceosome, JAK-STAT signaling pathway, ribosome, and Toll-like receptor signaling pathway were enriched (Fig. 9b). The target genes of lncRNA antisense, cis, and trans-regulation were mainly enriched in the notch signaling pathway, the glycosphingolipid biosynthesis-lacto complement, and spliceosome, respectively (Additional file 12: Figure S2). In the circRNAs, the MAPK signaling pathway, protein processing in the endoplasmic reticulum, and ubiquitin-mediated proteolysis were identified as predominantly enriched KEGG pathways (Fig. 9c).

### Target predictions and lncRNA-mRNA and circRNA-miRNA-mRNA regulatory network analysis

**Fig. 10:**
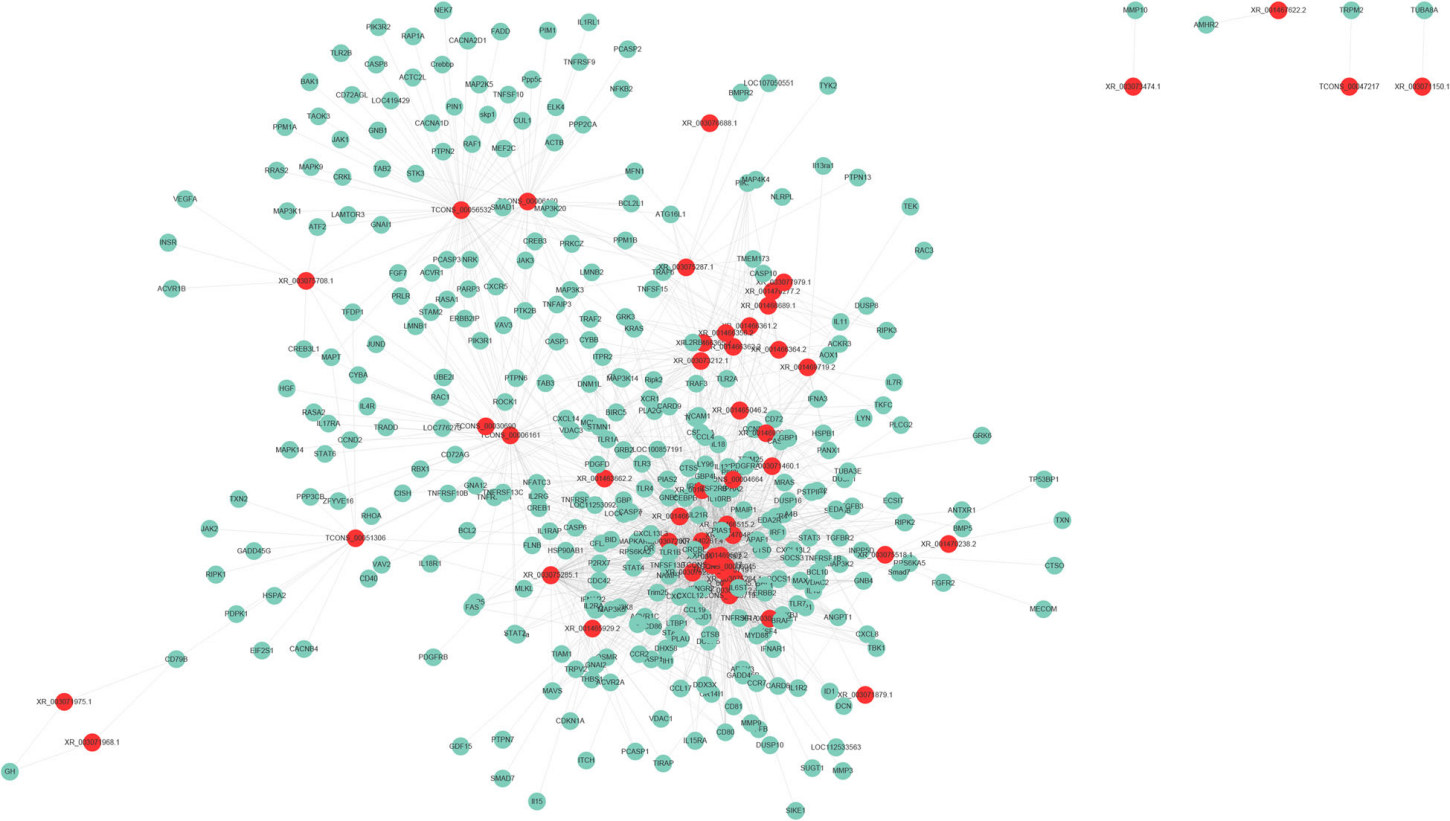
LncRNA-mRNA regulatory networks. The red ellipses indicate the differentially expressed lncRNAs, and the green ellipses indicate the differentially expressed mRNAs

Generally, lncRNAs may exert their effects by regulating their target genes. In the study, we predicted the potential target genes of lncRNAs and constructed lncRNA-mRNA regulatory networks (Fig. 10). A total of 2101 pairs of lncRNA-target genes containing 51 lncRNAs and 342 mRNAs (Additional file 13) were detected. In the lncRNA-mRNA network, LOC112532624 (XR_003075708.1) with the most significant difference (fold change = 703.3). Moreover, LOC107053928 (XR_001467739.2), LOC107054815 (XR_001469507.2), LOC107053352 (XR_001466515.2), and LOC107053557 (XR_001466920.2 97) was predicted to regulate the expression of 110, 100, 100 and 97 target genes, respectively, involved in antiviral responses, including *STAT1*, *STAT3*, *STAT4*, *TRIM25*, and *IFIH1* (Additional file 14: Table S3). More importantly, a total of 44 differentially expressed lncRNAs were found to be co-expressed with *STAT1*, a key antiviral marker molecule. The target prediction indicated that a lncRNA had multiple target genes, and a target gene was also targeted by multiple lncRNAs.

**Fig. 11:**
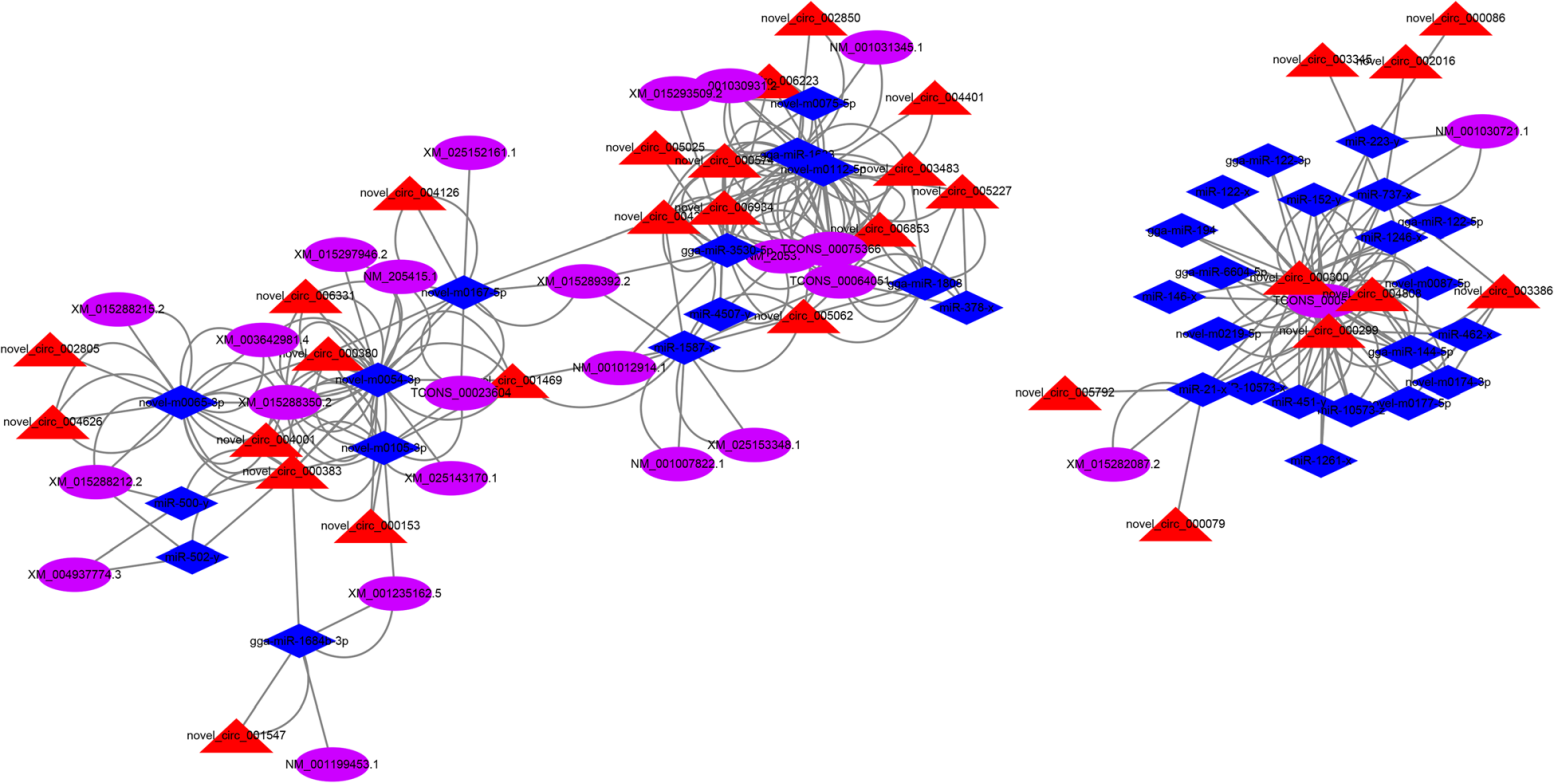
CircRNA-miRNA-mRNA regulatory networks. The red triangles, blue diamonds, and purple ellipses indicate the differentially expressed circRNA, miRNAs, and mRNAs, respectively

Recent studies evidenced that circRNAs commonly play an important regulatory role as miRNA sponges in several diseases [16, 25–28]. Here, the potential miRNA targets of 30 differentially expressed circRNAs were predicted based on complementary base pairing, with 36 miRNAs identified (Fig. 11; Additional file 15). In the circRNA-miRNA-mRNA network, circRNAs would indirectly regulate 25 chicken mRNAs, such as *STAT1/4* and *IRF1/7*, indicating that these circRNAs might play a critical role in regulating vvIBDV-infection.

### Validation of differential gene expression by quantitative PCR

**Fig. 12:**
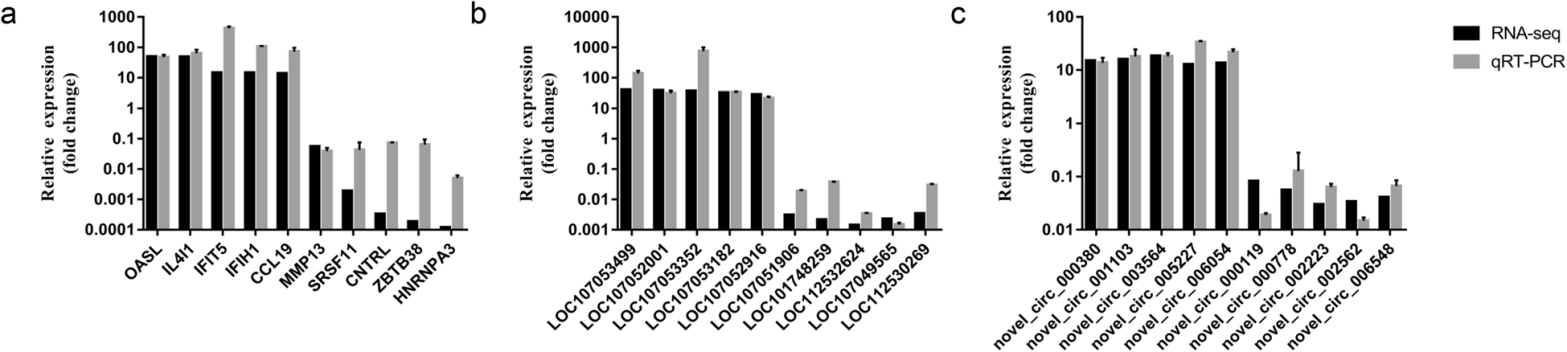
Validation of the differentially expressed mRNAs (**a**), lncRNAs (**b**), and circRNAs (**c**) by RT-qPCR. RT-qPCR experiments were performed in triplicate

To validate the accuracy of the RNA-sequencing results, 10 differentially expressed mRNAs, lncRNAs, and circRNAs were selected and quantified by reverse-transcription quantitative PCR (RT-qPCR; Fig. 12); these RT-qPCR results showed trends similar to those of RNA sequencing.

## Discussion

lncRNAs and circRNAs has emerged as regulator of gene expression and play important roles in various diseases [29, 30]. Many lncRNAs and circRNAs of chicken have been identified and analyzed by high-throughput sequencing technology, and suggesting that aberrantly expressed lncRNAs and circRNAs in cells or tissues play a crucial role in virus-host interactions [31–34]. Interesting, classical IBDV infection affects lncRNAs expression in DF-1 cell has been testified [11]. Infectious bursal disease caused by IBDV is one of the most important infectious diseases that severely affect the poultry industry. Infection with vvIBDV strains results in high mortality in chickens, and vvIBDV appears to have emerged as the predominant clinical disease type in nearly all poultry-producing regions of the world. Therefore, the BF of experimentally vvIBDV-infected chickens and control individuals were collected to study the differential expression profiles of mRNAs, lncRNAs, and circRNAs. In this study, 411 mRNAs, 272 lncRNAs, and 143 circRNAs were considered differentially expressed. The results indicated that these DEGs may play a significant role in the vvIBDV infection process, suggesting that they may include potential therapeutic targets for treating IBDV infections.

Host cells counteract the invasion of viral particles through a cascade of mechanisms, and IBDV infection elicits the expression of various genes. Previous study reported that the expression of interferon regulatory factor 8 (*IRF8*), *TRIM25*, *IFIT1*, *Mx1*; of *STAT1*, *STAT4*, and of Toll-like receptors, including *TLR3* and *TLR7*, is increased in IBDV-infected chickens or DF-1 or DT40 cells [4, 7, 11, 35–38]. In our study, the JAK-STAT signaling pathway, the NOD-like receptor signaling pathway, the cytokine-cytokine receptor interaction signaling pathway, apoptosis, the chemokine signaling pathway, and the Toll-like receptor signaling pathway were significantly enriched according to the KEGG enrichment analyses of the respective differentially expressed mRNAs. IBDV exploits these pathways to induce the expression of *STAT1*, *STAT3*, *STAT4*, *TRIM25*, *IFIT1*, and *Mx1* in the bursal tissue, and our results were in line with those of previous studies. In *STAT1*, a member of the STAT family, phosphorylation induces the expression of interferon-stimulating genes through a series of signal transduction steps to elicit antiviral mechanisms [39, 40]. Interestingly, *STAT1* can be regulated by many differentially expressed lncRNAs, suggesting that *STAT1* may be an important regulator during IBDV infection in chickens. Additionally, *STAT3*, *STAT4*, *TRIM25*, *IFIT1*, and *Mx1* may be involved in interactions between vvIBDV and the host.

The potential functions of lncRNAs are commonly predicted according to their target genes. Increasing evidence suggests that lncRNAs have important roles in adaptive or innate immune responses [41–43]. In a previous study, we found that loc107051710 suppressed the replication of IBDV by upregulating type I interferon production [11]. In the current study, it is worth noting that LOC107053928, LOC107054815, LOC107053352, and LOC107053557 were identified as regulated various target genes associated with immunomodulation, including *STAT1*, *STAT3*, *STAT4*, *TRIM25*, and *IFIH1*. TRIM25 is a member of the tripartite motif family of E3 ubiquitin ligases and has been demonstrated to play an important role in RIG-I antiviral pathways. Reportedly, TRIM25 can promote transcriptional upregulation of type I interferons (IFNs) by binding viral RNA-activated RIG-I [44, 45]. It has been well established that type I IFNs plays a crucial role in the antiviral processes, where type I IFNs can activate the STAT1 phosphorylation after binding its ligand to induce interferon-stimulating genes (ISGs) expression, such as *Mx1*, *OAS*, and *IFIH1*, through the JAK-STAT signaling pathway [40, 46]. Therefore, this implied that these lncRNAs and their target genes, *STAT1*, *STAT3*, *STAT4*, *IFIH1* and *TRIM25*, might play a vital role in the IBD anti-viral response. Intriguingly, gene targeting studies have shown that STAT1 target genes can promote antagonizing proliferation and inflammation; however, STAT3 was the opposite [47, 48]. Therefore, the expression levels of STAT1 and STAT3 may reflect the balanced response of the organism. We believe that further insight into the roles of LOC107053928, LOC107054815, LOC107053352, and LOC107053557 is very important for understanding the complex regulatory mechanism of gene expression in response to vvIBDV infection in chicken BF.

CircRNAs, a newly discovered class of ncRNAs, can affect the prognosis of diseases, especially tumors [49, 50] by regulating the activity of target genes and by participating in the regulation of gene transcription and protein production [51, 52]. In the current study, 63 upregulated and 80 downregulated circRNAs were identified, and their expression levels were generally lower than those of the mRNAs and lncRNAs. Most circRNAs in normal and cancer tissues in humans also occur at low abundance and may thus be by-products of pre-mRNA splicing [53, 54]. Additionally, circRNA can act as a competing endogenous RNA (ceRNAs) that impair miRNA activity through sequestration, thereby upregulating miRNA target gene expression [55]. In the study, circRNA-miRNA-mRNA network was constructed and showed that the regulatory relationships between circRNAs, miRNAs and target mRNAs were complex. In the network, circRNAs novel_circ_000574 and novel_circ_001469 were expressed 131,072-fold and 286,862-fold higher levels, respectively, during IBDV infection. Their potentially affected target genes involved in immune-related included *STAT1* and *IRF7* by binding to miR-1587-x and miR-4507-y respectively, suggesting that these circRNAs also play an important role in IBDV pathogenicity.

## Conclusion

Many signaling pathways were found to be involved in IBDV infection, particularly the JAK-STAT signaling pathways; however, further research is needed to assess their effects on the pathogenesis of IBDV infection. Additionally, we constructed lncRNA-mRNA and circRNA-miRNA-mRNA co-expression networks and predicted that LOC107053928, LOC107054815, LOC107053352, and LOC107053557 may affect viral replication by regulating *STAT1*, *STAT3*, *STAT4*, *IFIH1* and *TRIM25* expression. Our results provide new insights into the pathways and mechanisms that mediate host immune responses to vvIBDV. Further studies are needed to explore the biological functions of LOC107053928, LOC107054815, LOC107053352, and LOC107053557 during vvIBDV infection.

## Methods

### Study animals and viruses

Three-week-old specific-pathogen-free (SPF) White Leghorn chickens were obtained from the Harbin Institute of Veterinary Medicine (Harbin, China). A vvIBDV strain isolated from chicken BF and maintained in our laboratory was used in this study (strain LJ-5).

### Sample collection and preparation

Eighteen chickens were randomly assigned to two groups (control group and vvIBDV infection group) with nine individuals per group, and the two groups were housed independently. Chickens in the control group were injected with phosphate-buffered saline (PBS; PH = 7.4), and the vvIBDV infection group chickens were inoculated with the LJ-5 strain virus at a dose of 10^3^ × 50% embryo lethal dose (ELD) per 0.2 mL via eye-nose drops [56, 57]. The chickens were housed at an animal facility under pathogen-free conditions and were provided a standard diet and water. Moreover, these chickens were observed 2–3 times daily. On the third day after inoculation, three chickens from each group were randomly selected and euthanized via T-61 intravenously (0.4 ml/kg) to isolate the BF. The animal remains were treated innocuously according to the requirements of school animal welfare management. The procedures used in this experiment were approved by the Institutional Animal Care and Use Committee of Northeast Agricultural University (2016NEFU-315, 13 April 2017). All sections of this report adhere to the ARRIVE Guidelines for reporting animal research (Additional file 16). The National Centre for the Replacement, Refinement, and Reduction of Animals in Research (NC3Rs) is an independent scientific organization that minimize the use of animals in research and improve animal welfare and help overcome the challenges and limitations of the use of animals in research to the benefit of the whole bioscience community [58]. Samples were placed in liquid nitrogen and stored at − 80 °C. All instruments were treated with DEPC before use to remove RNases.

### RNA isolation, library preparation, and sequencing

Total RNA was isolated using Trizol reagent (Life Technologies, Carlsbad, USA), and the purity and integrity of the RNA were measured using a Nanodrop micro-spectrophotometer (Thermo Fisher Scientific, Waltham, USA) and an Agilent 2100 Bioanalyzer (Agilent Technologies, Santa Clara, USA), respectively. RNA samples qualified for analyses under the following conditions: concentration ≥ 100 ng/μL, RNA integrity number ≥ 7.0, and RNA 260/280 ratio of 1.8–2.0. For RNA sequencing, 3 μg RNA per sample was used, and ribosomal RNA was removed to generate sequencing libraries using a NEBNext® Ultra™ RNA Library Prep kit (NEB E7490L; New England Biolabs, Ipswich, USA). First-strand complementary DNA was synthesized using random hexamer primers and ProtoScript II Reverse Transcriptase, and the second strand was generated using a Second Strand Synthesis Enzyme Mix. Uracil N Glycosylase was used to digest complementary DNA, and Agencourt AMPure XP Beads (Beckman Coulter, Brea, USA) were used to purify library fragments to retain DNA fragments of 150–200 bp. The quality of the libraries was evaluated using a High Sensitivity DNA assay Kit (Agilent Technologies), and the libraries were sequenced on an Illumina HiSeq 4000 platform by a commercial service provider (Gene Denovo Biotechnology Co.; Guangzhou, China). For circRNA sequencing, RNase R (EPICENTRE Biotechnologies, Madison, USA) was used on rRNA-depleted RNAs to remove linear RNAs before sequencing library preparation.

### Filtration of raw sequencing reads

Raw reads containing adapters or bases of low quality affect assembly and analysis; therefore, to obtain clean high-quality sequences, raw reads were filtered to remove adapters, low-quality reads, and poly-N reads. Clean high-quality data was used for further analyses.

### RNA sequence mapping and transcriptome assembly

The short reads alignment tool Bowtie 2 (v. 2.2.8) [59] was used to map the sequences using an rRNA database. After removing the rRNA reads, the sequences were mapped to the *Gallus gallus* GRCg6a reference genome using TopHat2 (v. 2.1.1) [60]; Cufflinks (v. 2.1.1) [22] was used to assemble mapped reads independently with a reference annotation-based transcript assembly technique. Raw sequencing data were made available in the NCBI short reads archive.

### lncRNA and circRNA predictions

CNCI (version: 2.0) [61] and CPC [62] were used to assess the potential protein-coding functions of novel lncRNAs. Transcripts were mapped using the SwissProt database to assess protein annotation. The intersection of the respective results was chosen as lncRNAs. Phast (v. 1.3) [63], phyloFit [64], and PhastCons [65] were used to evaluate sequence conservation in the transcripts, calculate phylogenetic models among species, and compute the conservation scores of coding genes and lncRNAs, respectively. For circRNA, unmapped reads were extracted from the above results, and the ends of the unmapped reads were intercepted (default 20 bp) to identify the anchor reads, which were then mapped to the reference genome. The results were processed using find_circ software to predict circRNAs.

### Prediction of lncRNA and circRNA target genes

lncRNAs are involved in many post-transcriptional regulation processes, as are some other small RNAs such as microRNA (miRNA), the regulation of which depends on complementary base pairing. Some antisense lncRNAs may regulate gene silencing, transcription, and mRNA stability. To assess the interactions between lncRNAs and mRNAs, RNAplex software [66] was used to predict the complementary correlation of antisense lncRNAs and mRNAs. This software program includes the Vienna RNA package, and best base pairing predictions were based on a calculation of minimum free energy in the thermodynamic structure. Moreover, the cis and trans target genes of the differentially expressed lncRNAs were predicted. For the cis target genes, the mRNAs and the genomic location of the lncRNAs were mapped. We searched coding genes less than 100 kb up- or downstream of each lncRNA and then analyzed their functions. For the trans targets, correlations between the lncRNA and protein-coding gene expression was analyzed using Pearson’s correlation coefficient, and protein-coding genes with absolute correlation values > 0.94 were screened. The differentially expressed lncRNAs (fold change > 1.5 and *p* < 0.05) and predicted target genes (fold change > 2 and *p* < 0.05) were chosen to construct a co-expression regulatory network, which was visualized using Cytoscape software (v3.6.0) [67]. The binding sites of the miRNAs on the circRNAs and mRNAs were predicted using mirTarBase software [68], and the target relationships of miRNAs-mRNAs and miRNAs-circRNAs were assessed accordingly. Subsequently, we synthesized targeted relationships between the miRNAs and mRNAs and between the miRNAs and circRNAs to identify the mRNAs associated with the circRNAs.

### Functional annotation of DEGs

To understand the functions of the differentially expressed transcripts, including the mRNA and the target genes of the lncRNAs and circRNAs, the GO (http://www.geneontology.org/) and KEGG (http://www.kegg.jp/kegg/) databases were used to perform GO and KEGG enrichment analyses, respectively. GO terms and KEGG pathways with *p* values < 0.05 were considered significantly enriched.

### RT-qPCR validation of the RNA-sequencing results

To assess the accuracy of the sequencing results, five upregulated and five downregulated mRNAs, lncRNAs, and circRNAs were selected and quantified by RT-qPCR. Primers were designed using oligo6 software (Tables 4, 5 and 6). Total RNA was extracted from vvIBDV-infected and uninfected bursal tissue. RT-qPCR was performed using SYBR Green Master (Roche, Mannheim, Germany) and an ABI 7500 Real-Time PCR system (Applied Biosystems, Waltham, USA). β-Actin was used as an internal control of the mRNA. The 2^−ΔΔCt^ method was used to calculate the relative expression levels of the target genes. Experiments were conducted using three replicates.

**Table 4:** RT-qPCR primers used for verification of mRNA results

Gene	Primer sequence (5′-3′)	Accession number
β-actin	F-TGCTGTGTTCCCATCTATCG	X00182
	R-TTGGTGACAATACCGTGTTCA	
OASL	F-TCGGAGTCAGCATCACCAGTCC	NM_205041
	R-GCGTCGTAAGCAGGCAGGATG	
IL4I1	F-GCCTGGTACTTCGTGAACG	XM_015294015
	R-GAACTCCTTGACAGCCGAC	
IFIT5	F-CCCTCTCAAGCTGAAGCACT	NM_001320422
	R-TGAACAGACAAGCAAACGCA	
IFIH1	F-TGAAGAAAGGCGGCTGTGAC	NM_001193638
	R-GCACACAGAGATCGTGGTCG	
CCL19	F-TGCCTTAGTCTCCTGGTGCT	NM_001302168
	R-GCTGCATCCTGTAGTCCTGC	
MMP13	F-ACGCCAGAGAAATGTGCTGC	NM_001293090
	R-TCTGCTTCAACCATCTGCGG	
SRSF11	F-GGCCCAGCATCTGACAAACA	XM_015291055.2
	R-AGCTGGTGCCAAGAGAGACA	
CNTRL	F-TCAGCAGCACTTCCTCAGACTCC	XM_025156053
	R-TGTCCTGTCAGAGCAGCCTGTG	
ZBTB38	F-GAGACATGAAGACTCGGCTGTGAC	XM_015276974.2
	R-CAACGTACTGGCTGGCTCTGC	
HNRNPA3	F-GTGGTGGATATGGAGGTGGAGGAG	XM_004942715.3
	R-GGACCGTAATTGGACTGCTGCTG

**Table 5:** RT-qPCR primers used for verification of lncRNA results

Gene	Primer sequence (5′-3′)	Accession number
LOC107053499	F-TAGCCGTGAGCCCTGAGTTGG	XR_001466768.2
	R-AGCGATGTGGCAGCGGATTG	
LOC107052001	F-GCAGCGTCTCCAAACAGAAGGG	XR_001464039.2
	R-GTGAAAGCGAGTGGGGTTGAGG	
LOC107053352	F-GGCTGCTGCTCAGTGTCTCATG	XR_003071155
	R-CCCACCTCATCCCACCATCCTC	
LOC107053182	F-TGCCAACCCTGTGAAGATTGCC	XR_001466217.2
	R-GTGGGGAAGCAGCAGGTTTGTC	
LOC107052916	F-AGATGCTGGCAACTACACCTG	XR_001465729.2
	R-CATTTGCCCATTGGAGTCTAC	
LOC107051906	F-GAGCCCTGCTTGGGACCTTTTG	XR_001463887.2
	R-AATGCTGGTGCGTGAGTGAGTG	
LOC101748259	F-TGGCTCCTGTCGCTGTCCTC	XR_001470273.2
	R-ACGGCACCACAGAACAGTGTAAC	
LOC112532624	F-TTGCGAGCAGCGATTACTGAGAG	XR_003075708
	R-GGCAGATACAGAGTTGGACAAGGC	
LOC107049565	F-TGCTGGTGAGGAGGCTGAGATG	XR_003075803
	R-TGGCAGAACAGGCAGTTGAAGAAG	
LOC112530269	F-ACTTGCTTTCCCGTGCTGTGG	XR_003071776
	R-CCGAAATGCTTGGGCGTTTGC

**Table 6:** RT-qPCR primers used for verification of circRNA results

Gene	Primer sequence (5′-3′)
novel_circ_000380	F-CAACTCTCTGACTCAAACTA
	R-AGGTCCCAATATACTTAGTAC
novel_circ_001103	F-GACACAAGAATCGTGTGACATT
	R-CTAAGCTCCGACGCTCAATGT
novel_circ_003564	F-CCTGCCTGACCTGCATCG
	R-GTGAGTGAACTGTCCAGGTCTG
novel_circ_005227	F-CAGTTGCACCTGCCAAGAG
	R-GGCAGTCAACCTTACCTTGAAC
novel_circ_006054	F-CTCCCAGTTCAACTCCGATGAC
	R-ACCTGGTCATTGTGCTCCTTCA
novel_circ_000119	F-AGTGTTGTTGAGTCCTCATGCA
	R-TGATGAACAGAGTGTTAATGGT
novel_circ_000778	F-CAGTATTTGTCACATGCTGAAG
	R-CTACATCGACAATCTTTACAGC
novel_circ_002223	F-TCTGATCCTGTAATACAAGTCT
	R-GGAACACATCTTGTAGAGATCT
novel_circ_002562	F-GTCTTCATGGGAGGAAGAATAT
	R-TTCTTCTTGATTTATGGCATTT
novel_circ_006548	F-CTCCTTCCGAGAGACCTCTT
	R-CGCTGTCGTCACTATGCGAG

### Statistical analyses

GraphPad Prism5 software was used to analyze the RT-qPCR results with one-way ANOVA. Data are shown as the means ± standard error of the mean. Statistical significance was considered at *p* < 0.05.

## Supplementary information

**Additional file 1: Table S1.** Data quality of lncRNA and mRNA profiles.

**Additional file 2: Table S2.** High quality clean reads compared with the ribosomal RNA.

**Additional file 3.** The information of all known lncRNAs identified in this study.

**Additional file 4.** The information of all novel lncRNAs identified in this study.

**Additional file 5.** The information of all novel circRNAs identified in this study.

**Additional file 6.** All the differentially expressed mRNAs in this study.

**Additional file 7.** All the differentially expressed lncRNAs in this study.

**Additional file 8.** All the differentially expressed circRNAs in this study.

**Additional file 9.** GO enrichment analysis of mRNAs.

**Additional file 10: Figure S1.** Gene ontology enrichment analysis for the antisense, cis, and trans roles of the differentially expressed lncRNAs in chicken BF between the two groups; **a** antisense; **b** cis; and **c** trans. The green, red, and blue columns indicate biological processes (BPs), cellular components (CCs), and molecular functions (MFs), respectively.

**Additional file 11.** KEGG pathway analysis of mRNAs.

**Additional file 12: Figure S2.** Kyoto Encyclopedia of Genes and Genomes pathway enrichment for the antisense, cis, and trans roles of the differentially expressed lncRNAs in chicken BF between the two groups; **a** antisense; **b** cis; and **c** trans. The vertical axis shows the pathways, and the horizontal axis indicates the Rich factor. The dot size indicates the number of differentially expressed genes in the pathway, and the coloration corresponds to the Q-value range.

**Additional file 13.** LncRNA-mRNA co-expression relationships.

**Additional file 14: Table S3.** The target genes of lncRNAs.

**Additional file 15.** CircRNA/mRNA-miRNA co-expression relationships.

**Additional file 16.** ARRIVE guidelines checklist.

## Data Availability

The National Center for Biotechnology Information (NCBI) Nucleotide database accession number for IBDV LJ-5 strain is MT133301 (A fragments: https://www.ncbi.nlm.nih.gov/nuccore/MT133301.1/) and MT446362 (B fragments: https://www.ncbi.nlm.nih.gov/nuccore/MT446362). The raw data sets supporting the results of this article are available in the NCBI short reads archive and accession number is PRJNA635782. For information linking and citing, please refer to: https://www.ncbi.nlm.nih.gov/search/all/?term=PRJNA635782.

## Abbreviations

IBDV: Infectious bursal disease virus
BF: Bursa of Fabricius
lncRNAs: Long non-coding RNAs
circRNAs: Circular RNAs
NC3Rs: The National Centre for the Replacement, Refinement, and Reduction of Animals in Research
DEGs: Differentially expressed genes
GO: Gene Ontology
KEGG: Kyoto Encyclopedia of Genes and Genomes
BP: Biological processe
CC: Cellular component
MF: Molecular function
IRF8: Regulatory factor 8
IFNs: Interferons
ISGs: Interferon-stimulating genes
ceRNAs: Competing endogenous RNA
miRNA: microRNA
