# Supplementary material for: Genome-wide analysis of differentially expressed mRNAs, lncRNAs, and circRNAs in chicken bursae of Fabricius during infection with very virulent infectious bursal disease virus
Source: BMC Genomics. 2020 Oct 19;21:724. doi: 10.1186/s12864-020-07129-1 (PMC7574500; doi:10.1186/s12864-020-07129-1)
Supplement: Supplementary file 1 — Additional file 1: Table S1. Data quality of lncRNA and mRNA profiles. [file 12864_2020_7129_MOESM1_ESM.docx]

**Table S1.** Data quality of lncRNA and mRNA profiles

| Sample | Raw reads | Clean Reads | Adapter (%) | Low quality (%) | Poly A (%) | N (%) |
| --- | --- | --- | --- | --- | --- | --- |
| CK-1 | 74111524 | 73379900 (99.01%) | 230874 (0.31%) | 499796 (0.67%) | 52 (0%) | 425 (0%) |
| CK-2 | 104058884 | 10315042 (99.13%) | 243040 (0.23%) | 664498 (0.64%) | 146 (0%) | 317 (0%) |
| CK-3 | 87501368 | 86619768 (98.99%) | 204420 (0.23%) | 676056 (0.77%) | 77 (0%) | 485 (0%) |
| LJ-1 | 92817248 | 91909516 (99.02%) | 219406 (0.24%) | 687382 (0.74%) | 96 (0%) | 376 (0%) |
| LJ-2 | 149743398 | 148420960 (99.12%) | 350724 (0.23%) | 970406 (0.65%) | 222 (0%) | 432 (0%) |
| LJ-3 | 81543920 | 80804426 (99.09%) | 189054 (0.23%) | 549704 (0.67%) | 71 (0%) | 297 (0%) |
