# Supplementary material for: Genome-wide analysis of differentially expressed mRNAs, lncRNAs, and circRNAs in chicken bursae of Fabricius during infection with very virulent infectious bursal disease virus
Source: BMC Genomics. 2020 Oct 19;21:724. doi: 10.1186/s12864-020-07129-1 (PMC7574500; doi:10.1186/s12864-020-07129-1)
Supplement: Supplementary file 2 — Additional file 2: Table S2. High quality clean reads compared with the ribosomal RNA. [file 12864_2020_7129_MOESM2_ESM.docx]

**Table S2.** High quality clean reads compared with the ribosomal RNA

| Sample | All Reads Num | Mapped Reads | Unmapped Reads |
| --- | --- | --- | --- |
| CK-1 | 73379900 | 9767374 (13.31%) | 63612526 (86.69%) |
| CK-2 | 103150420 | 589836 (0.57%) | 102560584 (99.43%) |
| CK-3 | 86619768 | 431666 (0.50%) | 86188102 (99.50%) |
| LJ-1 | 91909516 | 419074 (0.46%) | 91490442 (99.54%) |
| LJ-2 | 148420960 | 860884 (0.58%) | 147560076 (99.42%) |
| LJ-3 | 80804426 | 436058 (0.54%) | 80368368 (99.46%) |
