# Supplementary material for: Genome-wide analysis of differentially expressed mRNAs, lncRNAs, and circRNAs in chicken bursae of Fabricius during infection with very virulent infectious bursal disease virus
Source: BMC Genomics. 2020 Oct 19;21:724. doi: 10.1186/s12864-020-07129-1 (PMC7574500; doi:10.1186/s12864-020-07129-1)

**Fig. S1** Gene ontology enrichment analysis for the antisense, cis, and trans roles of the differentially expressed lncRNAs in chicken BF between the two groups; **a** antisense; **b** cis; and **c** trans. The green, red, and blue columns indicate biological processes (BPs), cellular components (CCs), and molecular functions (MFs), respectively.


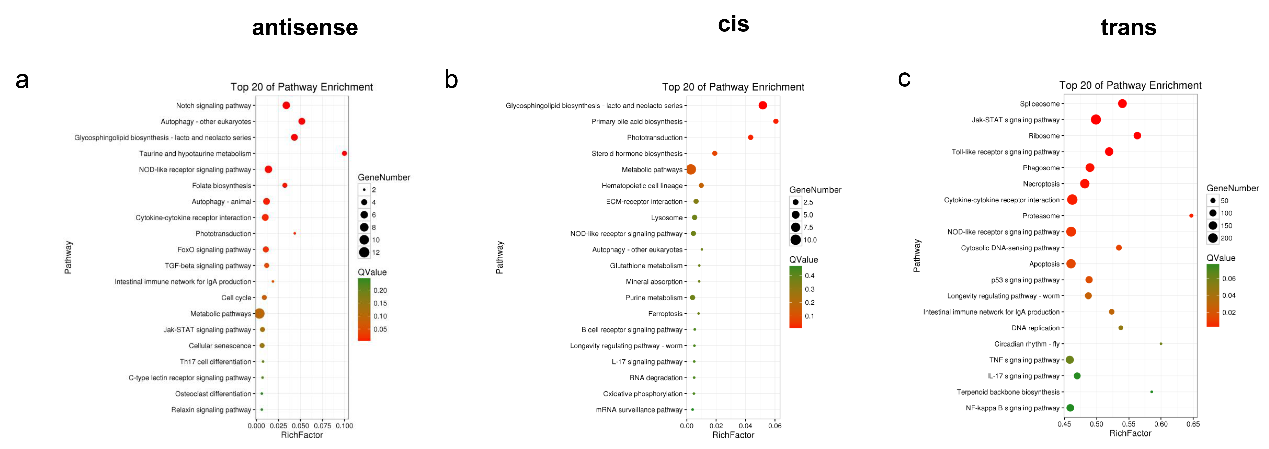

Supplement: Supplementary file 10 — Additional file 10: Figure S1. Gene ontology enrichment analysis for the antisense, cis, and trans roles of the differentially expressed lncRNAs in chicken BF between the two groups; a antisense; b cis; and c trans. The green, red, and blue columns indicate biological processes (BPs), cellular components (CCs), and molecular functions (MFs), respectively. [file 12864_2020_7129_MOESM10_ESM.docx]
