# Supplementary material for: Genome-wide analysis of differentially expressed mRNAs, lncRNAs, and circRNAs in chicken bursae of Fabricius during infection with very virulent infectious bursal disease virus
Source: BMC Genomics. 2020 Oct 19;21:724. doi: 10.1186/s12864-020-07129-1 (PMC7574500; doi:10.1186/s12864-020-07129-1)

**Fig. S2** Kyoto Encyclopedia of Genes and Genomes pathway enrichment for the antisense, cis, and trans roles of the differentially expressed lncRNAs in chicken BF between the two groups; **a** antisense; **b** cis; and **c** trans. The vertical axis shows the pathways, and the horizontal axis indicates the Rich factor. The dot size indicates the number of differentially expressed genes in the pathway, and the coloration corresponds to the Q-value range.


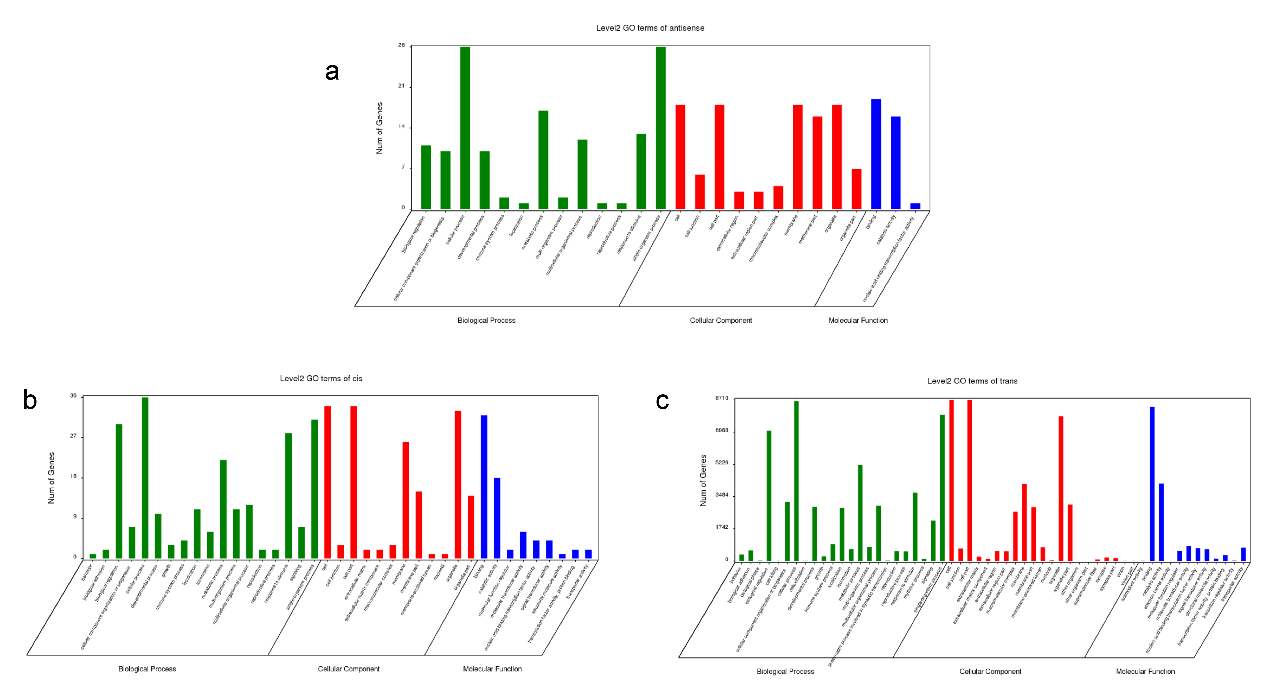

Supplement: Supplementary file 12 — Additional file 12: Figure S2. Kyoto Encyclopedia of Genes and Genomes pathway enrichment for the antisense, cis, and trans roles of the differentially expressed lncRNAs in chicken BF between the two groups; a antisense; b cis; and c trans. The vertical axis shows the pathways, and the horizontal axis indicates the Rich factor. The dot size indicates the number of differentially expressed genes in the pathway, and the coloration corresponds to the Q-value range. [file 12864_2020_7129_MOESM12_ESM.docx]
