# Supplementary material for: Genome-wide analysis of differentially expressed mRNAs, lncRNAs, and circRNAs in chicken bursae of Fabricius during infection with very virulent infectious bursal disease virus
Source: BMC Genomics. 2020 Oct 19;21:724. doi: 10.1186/s12864-020-07129-1 (PMC7574500; doi:10.1186/s12864-020-07129-1)
Supplement: Supplementary file 14 — Additional file 14: Table S3. The target genes of lncRNAs. [file 12864_2020_7129_MOESM14_ESM.docx]

**Table S3.** The target genes of lncRNAs

| lncRNA name | mRNA name |
| --- | --- |
| XR_001467739.2 | NOD1, LOC418667, IL10RB, MAPKAPK2, SOCS3, CCL4, PLAU, TNFRSF1A, IL10, TLR3, CD80, PSTPIP1, CD86, CXCL13L3, GBP1, RBL1, LY96, LTBP1, IFIH1, STAT1, IL6ST, ACKR4, ERBB2, LTBP1, Trim25, MAP3K5, TUBA4B, DDX3X, DHX58, EDA2R, GBP4L, IL13RA2, TNFSF4, DUSP5, IL10, DNM1L, CTSD, CASP1, P2RX7, STAT4, STAT4, CXCL13, PIAS1, MAP3K2, CCL19, IRF1, IL6ST, LY96, BRAF, BIRC2, CCR7, STAT1, DHX58, STAT3, IL18, IL1RAP, IL2RA, CARD9, KDR, CEBPB, IFNAR1, TLR3, MAP3K8, PMAIP1, LOC100857191, CD86, CD81, ANGPT1, DUSP16, EDA, CSF2RB, TUBA3E, IRF1, IFNAR2, STAT1, TGFB3, IL13RA2, CTSB, RIPK2, GBP, CXCL12, STAT3, TRIM25, CRCBL, CXCL13L2, IRF7, PDGFRA, RPS6KB1, PIAS2, CSF2RB, CCL17, CXCL12, CRCBL, MYD88, STAT3, TNFRSF1B, CCR2, SOCS1, TLR4, CTSS, NAMPT, TLR1B, CARD8, APAF1, CASP7, PDGFRA, EPHA2, NAMPT, CRCBL, CDC42 |
| XR_001466515.2 | NOD1, LOC418667, IL10RB, MAPKAPK2, SOCS3, CCL4, TLR3 ,PLAU, TNFRSF1A, RPS6KA2, IL10, TLR3, KRAS, GBP1, TRAF1, LY96, IFIH1, STAT1, IL6ST, ACKR4, ERBB2, LTBP1, Trim25, MAP3K5, TUBA4B, DDX3X, DHX58 ,EDA2R, GBP4L, IL13RA2, TNFSF4, DUSP5, DNM1L, CTSD, CASP1, P2RX7, STAT4, STAT4, CXCL13, PIAS1, CD72, CCND3, CCL19, IRF1, IL6ST, PANX1, LY96, BRAF, BIRC2, STAT1, DHX58, STAT3, Smad7, IL18, TGFBR2, CARD9, KDR, CEBPB, IFNAR1, MAP3K8, LOC100857191, CD86, CD81, ANGPT1, DUSP16, HSPB1, EDA, CSF2RB, IRF1, STAT1, IL18, TGFB3, IL13RA2, CTSB, CXCL12, STAT3, TRIM25, CRCBL, CXCL13L2, IRF7, PDGFRA, RPS6KB1, PIAS2, CSF2RB, CXCL12, CRCBL, MYD88, TNFRSF1B, CCR2, SOCS1, SP1, TLR4, NAMPT, TLR1B, CARD8, APAF1, CASP7, PDGFRA, NAMPT, CRCBL |
| XR_001469507.2 | RBL1, NOD1, IL10RB, MAPKAPK2, BID, SOCS3, PLAU, TNFRSF1A, RPS6KA2, FLNB, GNAI2, CD80, PSTPIP1, CD86, CXCL13L3, GBP1, STMN1, LY96, LTBP1, IFIH1, STAT1, IL6ST, ACKR4, ERBB2, LTBP1, Trim25, MAP3K5, TUBA4B, DDX3X, DHX58, EDA2R, GBP4L, IL13RA2, TNFSF4, DUSP5, IL10, CTSD, CASP1, P2RX7, STAT4, STAT4, CXCL13, PIAS1, CCL19, IRF1, IL6ST, LY96, BRAF, BIRC2, CCR7, STAT1, DHX58, STAT3, IL18, HSP90AB1, IL1RAP, CEBPB, IFNAR1, RPS6KB1, MAP3K8, PMAIP1, LOC100857191, CD86, ANGPT1, DUSP16, IL2RG, CSF2RB, PDGFD, IRF1, IFNAR2, STAT1, TGFB3, IL13RA2, CTSB, GBP, CXCL12, STAT3, TRIM25, MAVS, CRCBL, IRF7, STAT4, RPS6KB1, CSF2RB, CRCBL, MYD88, TNFRSF1B, CCR2, SOCS1, TLR4, CTSS, NAMPT, CARD8, APAF1, ADCY3, CASP7, PDGFRA, PXN, EPHA2, NAMPT |
| XR_001466920.2 | TIAM1, VCAM1, LOC418667, IL10RB, CDKN1A, SOCS3, CCL4, TLR3, PLAU, TNFRSF1A, TNFSF13B, TLR1A, RPS6KA2, IL10, TLR3, ACVR1C, CD80, LY96, STAT1, IL6ST, ACKR4, Trim25, MAP3K5, TUBA4B, EDA2R, CYBB, FAS, TNFSF4, DUSP5, DNM1L, Ripk2, CTSD, MLKL, P2RX7, STAT4, STAT4, CXCL13, IFNA3, CD72, ITPR2, BIRC5, CCL19, BRAF, BIRC2, CCR7, CSF2RA, DHX58, CD86, IL1RAP, IL2RA, TGFBR2, PLA2G4A, CARD9, KDR, CEBPB, IFNAR1, LOC100857191, CD86, ANGPT1, DUSP16, CSF2RB, IFNAR2, STAT1, IL18, TGFB3, IL13RA2, CTSB, IL18, RIPK2, TRIM25, CXCL13L2, IRF7, TLR2A, PDGFRA, RPS6KB1, PIAS2, CSF2RB, TNFRSF11A, IL2RB, CRCBL, MYD88, TNFRSF1B, CCR2, SOCS1, TLR4, THBS1, NAMPT, VCAM1, CSF1R, APAF1, GRK3, CASP7, PDGFRA, VAV3, NAMPT, CRCBL, CDC42 |
