# Supplementary material for: Genome-wide analysis of differentially expressed mRNAs, lncRNAs, and circRNAs in chicken bursae of Fabricius during infection with very virulent infectious bursal disease virus
Source: BMC Genomics. 2020 Oct 19;21:724. doi: 10.1186/s12864-020-07129-1 (PMC7574500; doi:10.1186/s12864-020-07129-1)

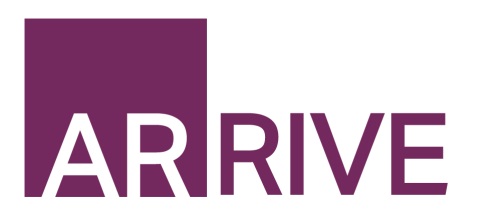


The ARRIVE Guidelines Checklist

Animal Research: Reporting In Vivo Experiments

Carol Kilkenny^1^, William J Browne^2^, Innes C Cuthill^3^, Michael Emerson^4^ and Douglas G Altman^5^

*^1^The National Centre for the Replacement, Refinement and Reduction of Animals in Research, London, UK, ^2^School of Veterinary Science, University of Bristol, Bristol, UK, ^3^School of Biological Sciences, University of Bristol, Bristol, UK, ^4^National Heart and Lung Institute, Imperial College London, UK, ^5^Centre for Statistics in Medicine, University of Oxford, Oxford, UK.*

|  | | ITEM | RECOMMENDATION | Section/ Paragraph |
| --- | --- | --- | --- | --- |
| 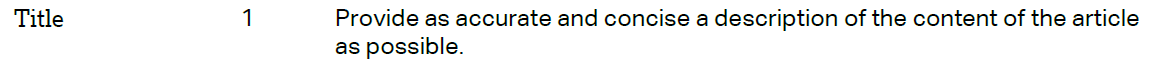 | | | Title |  |
| 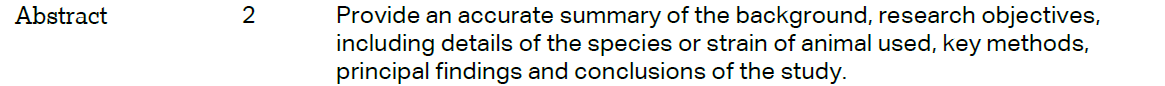 | | | Abstract |  |
| INTRODUCTION | | |  |  |
| 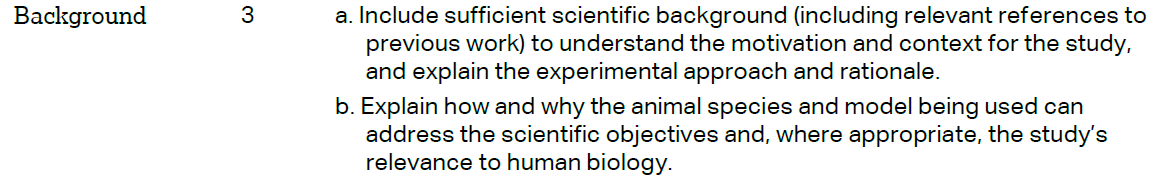 | | | Paragraphs 1-3  Paragraphs 1 & 3 |  |
| 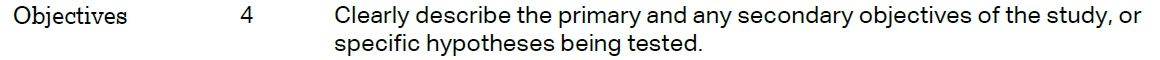 | | | Paragraph 3 |  |
| METHODS | | |  |  |
| 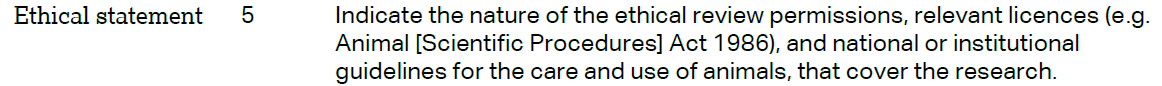 | | | Paragraph 2 |  |
| 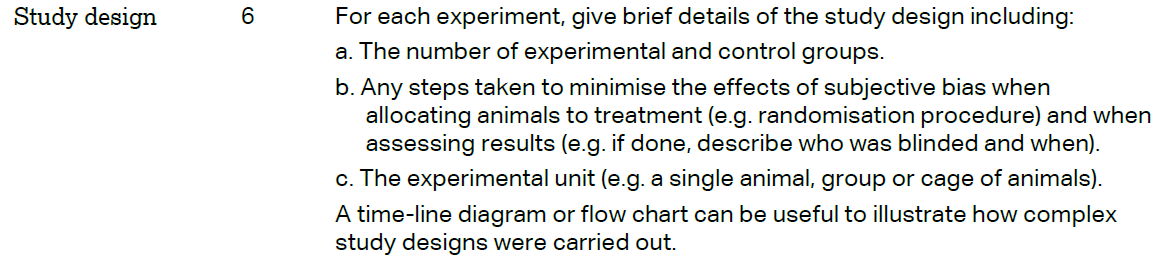 | | | Paragraph 2 |  |
| 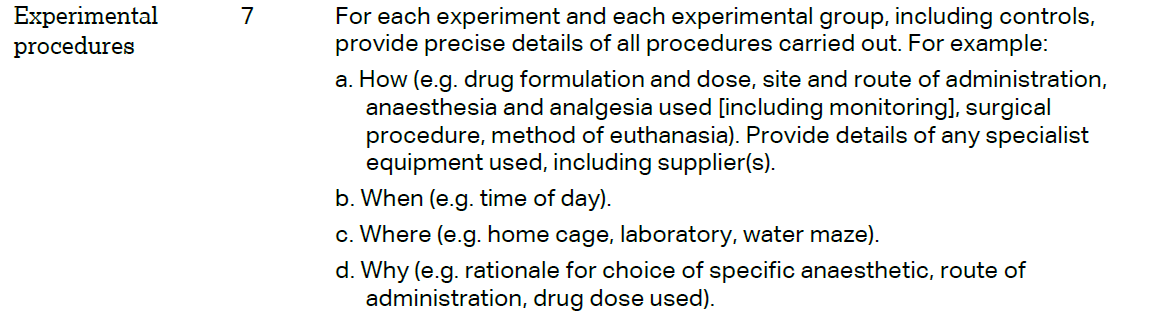 | | | Paragraph 2 |  |
| 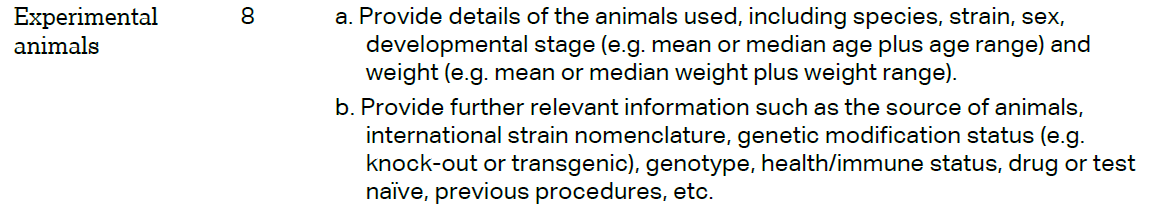 | | | Paragraph 1 |  |

The ARRIVE guidelines. Originally published in *PLoS Biology*, June 2010^1^

| 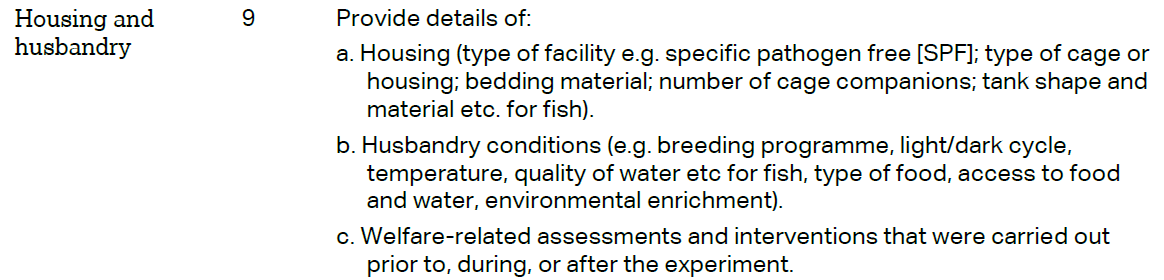 | Paragraph 2 |  |
| --- | --- | --- |
| 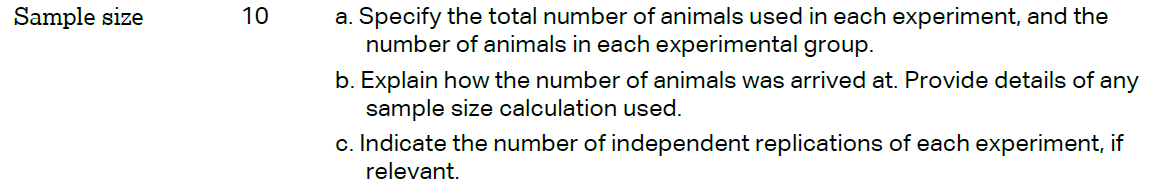 | Paragraph 2 |  |
| 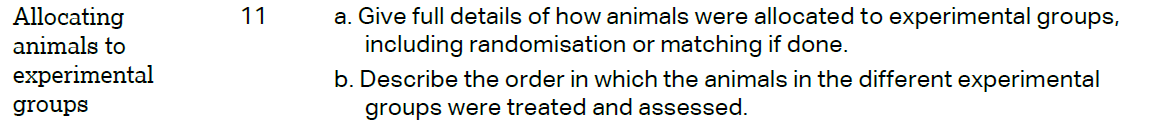 | Paragraph 2 |  |
| 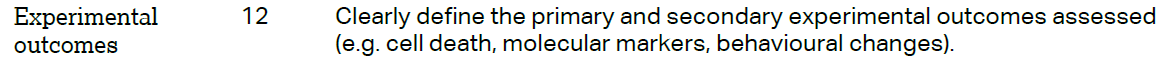 | Paragraphs 3 & 10 |  |
| 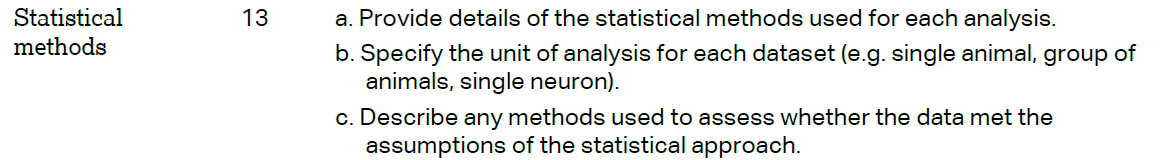 | Paragraph 10  Paragraph 2  Paragraph 3 |  |
| RESULTS |  |  |
| 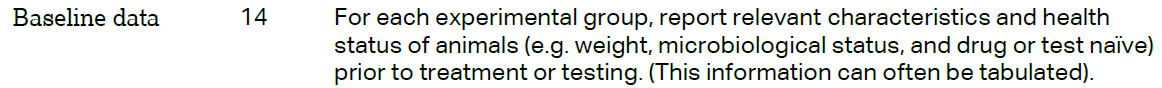 | Methods  paragraph 1 |  |
| 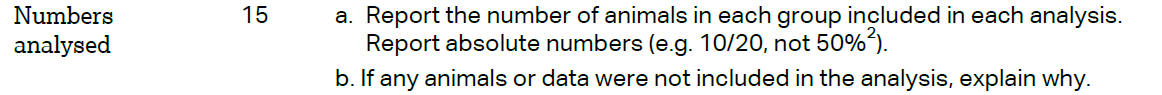 | Methods  paragraph 2 |  |
| 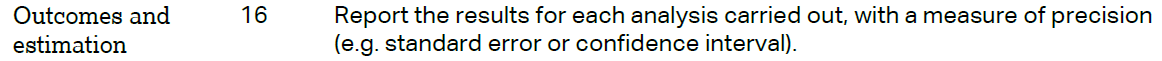 | Paragraphs 1, 2 and 3 |  |
| 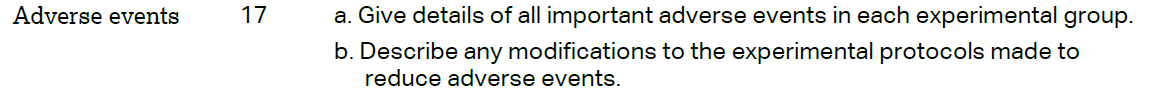 | Paragraph 2 |  |
| DISCUSSION |  |  |
| 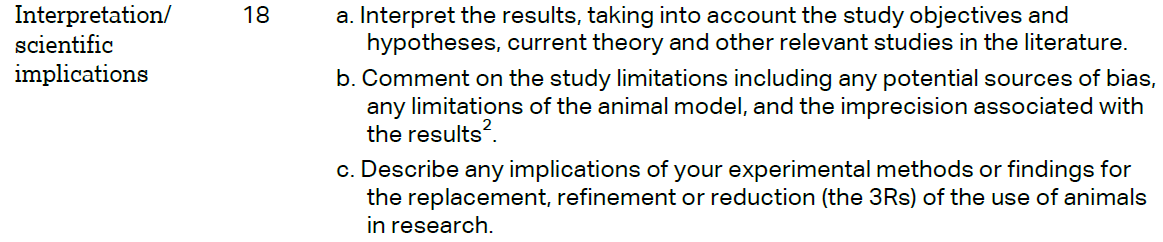 | Throughout  Paragraph 5  Methods  paragraph 2 |  |
| 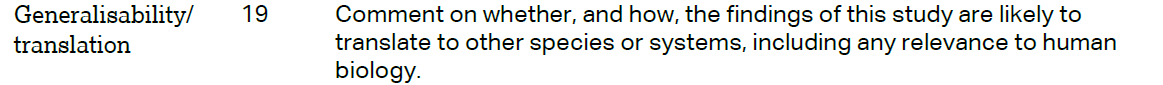 | Paragraph 5 |  |
| 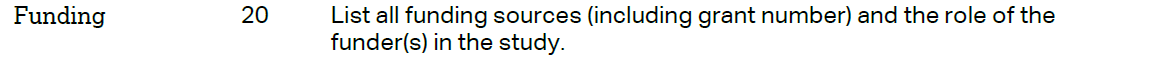 | | Funding |


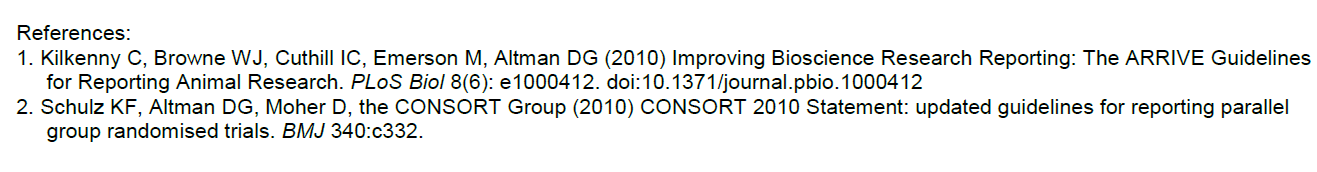

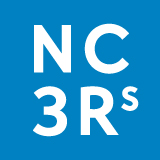

Supplement: Supplementary file 16 — Additional file 16. ARRIVE guidelines checklist. [file 12864_2020_7129_MOESM16_ESM.docx]
